# Felsic hydrothermal deposit formation by chalcophile metal concentration through mafic recharge and fluid-mediated sulfide dissolution

**DOI:** 10.1126/sciadv.aec4514

**Published:** 2026-07-15

**Authors:** Yifei Liu, Anthony E. Williams-Jones, Zengqian Hou, Leon Bagas, Sihong Jiang, Fengxiang Wang, Genyuan Ji

**Affiliations:** ^1^ State Key Laboratory of Deep Earth and Mineral Exploration, Institute of Mineral Resources, Chinese Academy of Geological Sciences, Beijing 100037, China.; ^2^ Department of Earth and Planetary Sciences, McGill University, Québec H3A 0E8, Canada.; ^3^ State Key Laboratory of Deep Earth and Mineral Exploration, Chinese Academy of Geological Sciences, Beijing 100037, China.; ^4^ Key Laboratory of Regional Geology and mineralization, HeBei GEO University, Shijiazhuang 050031, Hebei, China.

## Abstract

Hydrothermal sulfide deposits (e.g., porphyry and skarn) associated with felsic magmatism are major sources of chalcophile metals, including copper (Cu), molybdenum (Mo), gold (Au), lead (Pb), zinc (Zn), silver (Ag), and tin (Sn). However, the source of sulfur and metals remains debated. We report a study of clinopyroxene-hosted sulfide inclusions in mafic enclaves from the Beidashan granitic pluton (Northeast China), associated with a giant Sn-Pb-Zn-Ag-Cu deposit. Evidence shows the following: (i) immiscibility among Cu- and Zn-rich sulfide liquid, a silicate liquid, and an aqueous fluid during mafic magma emplacement in the upper crust; (ii) metal enrichment in FeS [iron(II) sulfide]–dominated melt inclusions (∼2628 parts per million of Cu, ∼233 parts per million of Zn, ∼275 parts per million of Pb, and ∼2.0 parts per million of Sn), reflecting strong partitioning into sulfide melts; and (iii) oxidative dissolution of sulfides by exsolved fluids, enabling metal transfer to the overlying felsic magma. The study demonstrates that metals in felsic-hosted deposits are sourced from subjacent mafic magmas and that mafic-felsic interaction triggered sulfide liquid/aqueous fluid cosaturation, ensuring efficient fluid-mediated chalcophile metal dissolution and transfer.

## Teaser

Sulfide-fluid cosaturation enables efficient, fluid-mediated transfer of metals from mafic to felsic hydrothermal systems.

## INTRODUCTION

Hydrothermal deposits associated with felsic rocks (e.g., porphyry, skarn, and epithermal vein deposits) are globally important sources of chalcophile metals, such as copper (Cu), molybdenum (Mo), gold (Au), lead (Pb), zinc (Zn), silver (Ag), and tin (Sn) (*1*). Nevertheless, aspects of their genesis remain enigmatic. For example, most chalcophile metals and sulfur have low solubility and abundance in felsic magmas, implying that the latter are not the sources of the metals needed to form the associated sulfide deposits (*2*–*7*). Some chalcophile metals, however, show crustal affinity because of their high solubility in felsic melts [Sn and W (tungsten)] or enrichment in alkali feldspar (Pb) (*8*–*10*). To reconcile the paradoxical co-occurrence of these metal groups in zoned polymetallic deposits, a hybrid model involving mantle-derived mafic and crust-derived felsic magmas is proposed (*11*).

Experimental studies show that chalcophile metals partition strongly into Cu-rich sulfide liquids relative to silicate liquids during mantle melting and magmatic differentiation. This applies to both low-solubility metals in felsic systems (e.g., Cu, Mo, Au, Zn, and Ag) and crustally compatible metals (e.g., Sn, W, and Pb) (*12*, *13*). This indicates sulfide-rich mantle-derived mafic magmas can supply both chalcophile metal categories. Moreover, aqueous volatiles facilitate the upward transfer of the metals from mafic magmas via fluid-mediated chemical dissolution (*14*–*16*). Early sulfide saturation in mafic systems may, nonetheless, lead to chalcophile metal depletion because of sulfide segregation [e.g., (*17*, *18*)], thereby reducing the potential for mineralization. Therefore, constraining the mechanisms and timing of the transfer of chalcophile-rich sulfides to felsic magmas is critical for resolving this issue.

The Beidashan granitic pluton in Northeast China, which was intruded at ∼141 to 138 million years (ma) in a postsubduction setting (*19*, *20*), provides an opportunity to gain new insights into the genesis of the zoned polymetallic magmatic-hydrothermal deposits referred to above. The pluton comprises two main lithologic units: a highly evolved, high-silica biotite alkali-feldspar granite and a quartz syenite that is rich in alkali feldspar phenocrysts and contains mafic enclaves. An ∼138-ma, K-spar (variation: amazonite) granite porphyry is also present in the pluton and is associated with a giant, reduced, low-sulfidation porphyry-epithermal ore system at Weilasituo. This ore system is systematically zoned in the distribution of its metals from proximal Sn through intermediate Cu-Zn to distal Pb-Zn-Ag veins, relative to the porphyry. The main sulfide minerals are pyrrhotite (po; ∼50%), iron (Fe)–rich sphalerite (sp; ∼15%), arsenopyrite (asp; ∼10%), pyrite (py; ∼5%), galena (gn; ∼5%), and chalcopyrite (ccp; ∼5%), and are accompanied by minor sulfosalts such as freibergite (*20*).

Here, we present petrological and geochemical evidence from clinopyroxene (cpx)–hosted sulfide-silicate melt inclusions within mafic enclaves that bear on the origin of the economic mineralization. This evidence demonstrates unequivocally that the chalcophile metals in the Weilasituo deposit were sourced from mafic recharge. It also shows that mafic-felsic magma interaction led to efficient metal transfer into the overlying felsic system through the near-coeval exsolution of a sulfide liquid and an aqueous fluid from the mafic magma.

## RESULTS

### Mafic enclaves and their sulfide inclusions

**Fig. 1.**
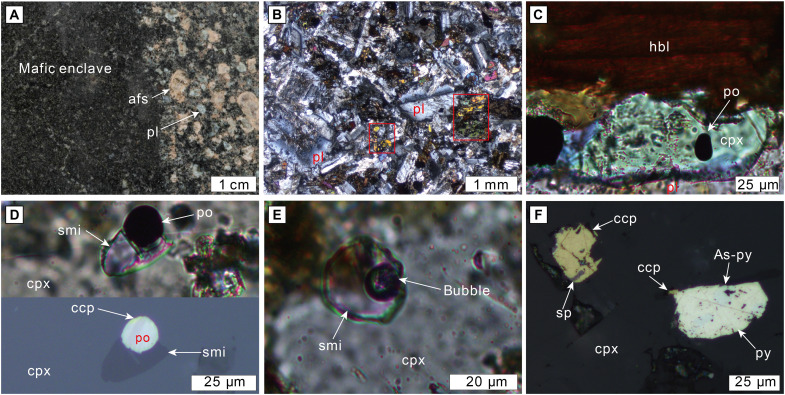
Petrographic photographs of the studied mafic enclaves. (**A**) Fine-grained mafic enclave quenched by quartz syenite containing alkali-feldspar (afs), (**B**) intergranular cpx (in the red rectangles) within a plagioclase (pl) matrix in a mafic enclave, (**C**) cpx partly altered to hornblende with a pyrrhotite (po) inclusion, (**D**) cpx with coentrapped pyrrhotite and high-silica melt inclusions [smi; a backscattered electron (BSE) image of the pyrrhotite inclusion is shown in Fig. 2A], (**E**) high-silica melt inclusion with a vapor bubble, and (**F**) chalcopyrite (ccp) crystal containing sphalerite (sp), pyrite (py), and As-rich pyrite (As-py) inclusions coentrapped in a cpx crystal, providing evidence of Cu-, Zn-rich sulfide-silicate immiscibility.

**Fig. 2.**
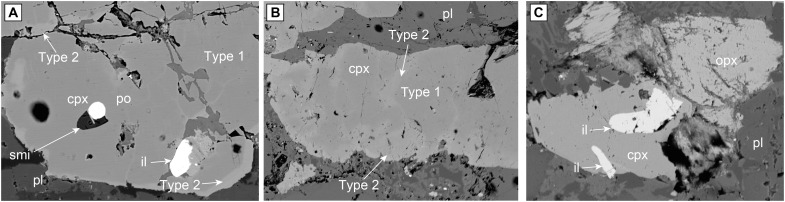
BSE images of pyroxene from mafic enclaves. (**A**) Intergranular cpx crystal containing pyrrhotite (po) and ilmenite (il) inclusions, a network of fine cracks, and evidence of replacement by a second generation of cpx; (**B**) early crystallizing coarse-grained cpx replaced by a second generation of cpx along its margins and along fractures within the crystal; and (**C**) coexisting intergranular cpx and orthopyroxene (opx), with the orthopyroxene showing significantly more evidence of intense alteration.

The mafic enclaves in the quartz syenite are spheroidal to irregular in shape, with sizes ranging from centimeters to decimeters (Fig. 1A). They are fine-grained with intergranular cpx in a matrix of equigranular (Fig. 1B) and smaller proportions of hornblende (hbl), orthopyroxene (opx), biotite, and ilmenite, and minor apatite (table S1). Microenclaves are also common and are dispersed with alkali feldspar phenocrysts in the quartz syenite (Fig. 1A). Large plagioclase and cpx crystals (up to 2 mm in diameter) occur locally in the enclaves and likely crystallized in the mafic magma before injection into the quartz syenite. Both the intergranular cpx and large cpx crystals (type 1) were replaced along their rims and along fractures within them by a second generation of cpx (type 2; Fig. 2, A and B). The cpx was subsequently altered partially to hornblende (Fig. 1C) and biotite; this alteration was more strongly developed in the orthopyroxene (Fig. 2C). These observations and evidence of quenched margins indicate that the enclaves represent mafic magma that crystallized rapidly and was injected into a crystal mush to form quartz syenite (*21*, *22*).

High-silica glassy inclusions [70.3 to 81.2 wt % silicon dioxide (SiO_2_); table S2) (*22*) measuring between 5 and 100 μm in diameter with a bubble up to 30 μm are ubiquitous in the intergranular cpx (Fig. 1, D and E) but are conspicuously absent in the coarse-grained cpx. Although such bubbles are commonly attributed to shrinkage during the cooling of the melt (*23*), Raman analysis shows clearly that some of the bubbles contain water (H_2_O) liquid and/or vapor (*22*). Sulfide inclusions are also common in the intergranular cpx as well as in hornblende, ilmenite, and plagioclase, locally co-occurring with glassy high-silica melt inclusions in cpx (Fig. 1D). However, they are not observed in the coarse-grained cpx.

**Fig. 3.**
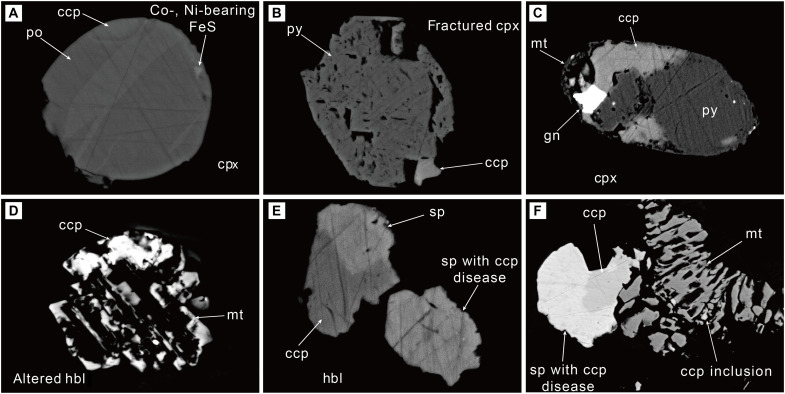
BSE images of sulfides from mafic enclaves. (**A**) Cpx-hosted pyrrhotite (po) inclusion containing tiny crystals of a Co-, Ni-bearing FeS phase and chalcopyrite (ccp) at its edge (Fig. 1D), (**B**) a porous pyrite inclusion with chalcopyrite at its margin in a fractured cpx crystal, (**C**) an oval sulfide inclusion in fractured cpx composed of pyrite (py) that was partly oxidized to magnetite (mt) and also contains chalcopyrite (ccp) and minor galena (gn), (**D**) a rounded sulfide inclusion in altered hornblende that was oxidized to porous magnetite containing chalcopyrite near its uppermost margin, (**E**) sulfide inclusions composed of chalcopyrite and sphalerite (sp) in altered hornblende, and (**F**) an intergranular inclusion of chalcopyrite and sphalerite and an inclusion oxidized to magnetite, containing relict chalcopyrite.

Sulfide inclusions in fresh, unfractured intergranular cpx are typically spherical to ellipsoidal in shape and ∼10 to 25 μm in diameter (Fig. 1D). They are dominated by pyrrhotite and commonly contain minor chalcopyrite near their margins. Locally, the spherical pyrrhotite inclusions contain tiny Co-Ni–bearing grains and coexist with glassy high-silica melt inclusions (Fig. 3A).

In contrast to the sulfide inclusions in the unfractured intergranular cpx, those within fractured intergranular cpx are composed mainly of pyrite instead of pyrrhotite and are commonly rimmed by chalcopyrite (Fig. 3B). Pyrite and chalcopyrite globules were identified in a single cpx crystal (Fig. 1F). In addition, sulfide globules containing chalcopyrite and galena, which have been partly oxidized to porous magnetite, were observed in the fractured intergranular cpx (Fig. 3C).

The sulfide globules in ilmenite, plagioclase, and altered hornblende are composed mainly of pyrite with a chalcopyrite rim. In some cases, they have been partly or completely oxidized to porous magnetite crystals, with chalcopyrite preserved at their edges (Fig. 3D). Sphalerite inclusions are common in hornblende that replaced cpx and are frequently associated with chalcopyrite inclusions (Fig. 3E).

Sphalerite, chalcopyrite, and galena occur as globules in the interstices between cpx, plagioclase, and hornblende crystals and have been oxidized and resorbed to porous magnetite. The porous magnetite contains inclusions of chalcopyrite intergrown with sphalerite and chalcopyrite, providing textural evidence for its formation via the oxidation of the iron in preexisting sphalerite and chalcopyrite (Fig. 3F). The sphalerite-to-chalcopyrite ratio in the globules is ∼8:1. Further detail on the modes of occurrence of the minerals in the sulfide globules is provided in table S1.

### Mineral composition and pressure-temperature conditions

**Fig. 4.**
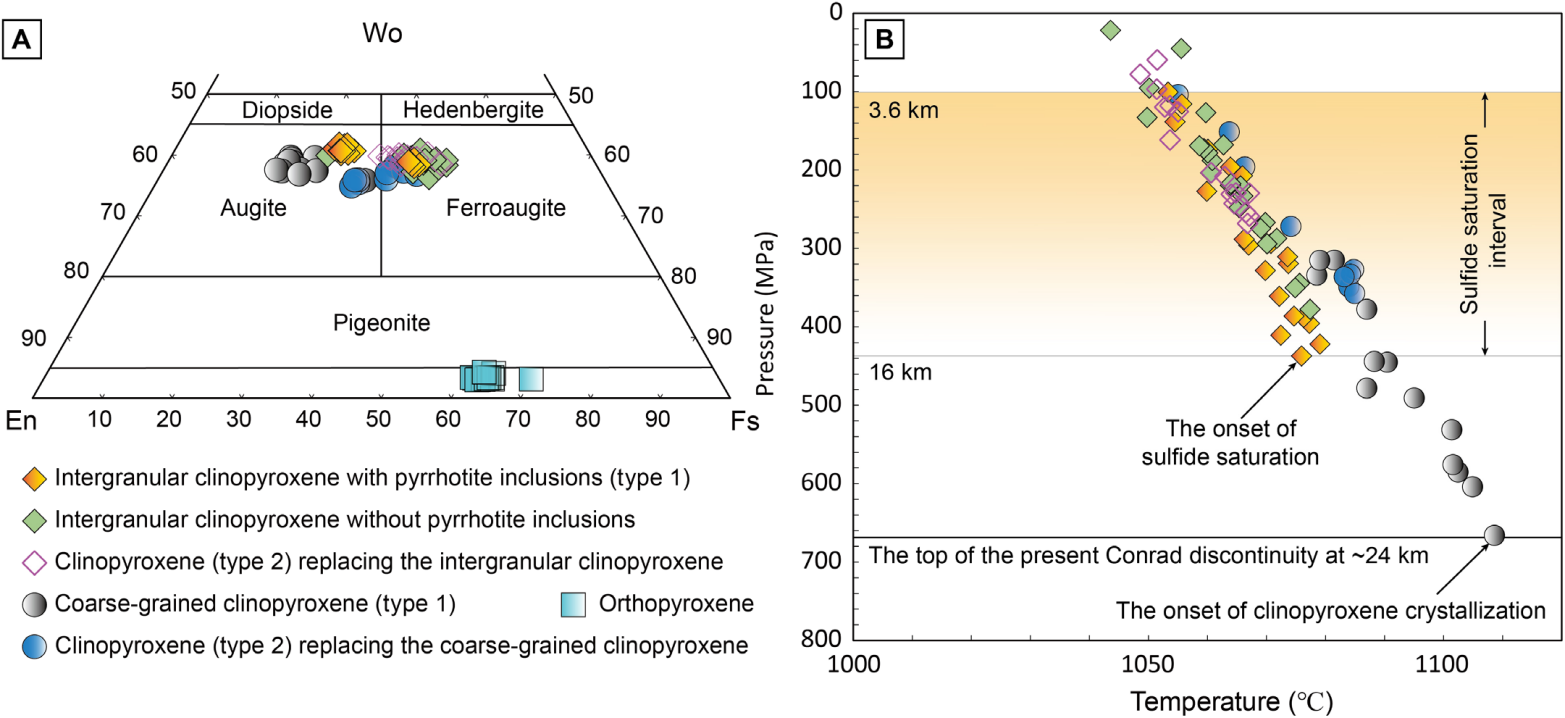
Composition and *P*-*T* conditions of pyroxene crystallization from mafic enclaves. (**A**) Pyroxene classification (*24*) and (**B**) the estimated *P*-*T* conditions of cpx crystallization. The coarse-grained cpx began crystallizing at ∼670 MPa (corresponding to a depth of ∼24 km), a pressure that coincides with the top of the present Conrad discontinuity [the mafic-felsic transition in the continental crust (*40*)]. Sulfide liquid saturation occurred at ∼440 MPa or a depth of 16 km and was triggered by mafic magma–induced melting of the felsic crust during ascent of the mafic magma.

The cpx displays a wide range of composition on a pyroxene classification diagram (Fig. 4A and table S3) (*24*). Early-crystallized, coarse-grained cpx has the highest enstatite (En) proportion, followed by intergranular cpx containing pyrrhotite inclusions, whereas the second-generation (replacement) cpx has the lowest En content. The type 1 cpx has a relatively low content of Cu [<0.9 parts per million (ppm)], Pb (<0.8 ppm), and As (<3 ppm) but a higher Zn content (∼105 ppm) (table S4).

Cpx-liquid thermobarometry was conducted using the major element composition of the different types of cpx and the bulk composition of the mafic enclaves (*25*, *26*). This revealed a progressive decrease in pressure-temperature (*P*-*T*) conditions from those of the crystallization of the coarse-grained cpx (∼670 to 320 MPa, ∼1109° to 1079°C), through those of intergranular cpx (∼440 to 100 MPa, ∼1079° to 1053°C), to the crystallization of the second-generation replacement cpx (∼270 to 60 MPa, ∼1067° to 1049°C) (Fig. 4B). Two-pyroxene thermometry on coexisting mineral pairs yielded crystallization temperatures of ∼1008° to 1058°C for the intergranular cpx, consistent with the results of cpx-liquid thermobarometry (Materials and Methods).

**Fig. 5.**
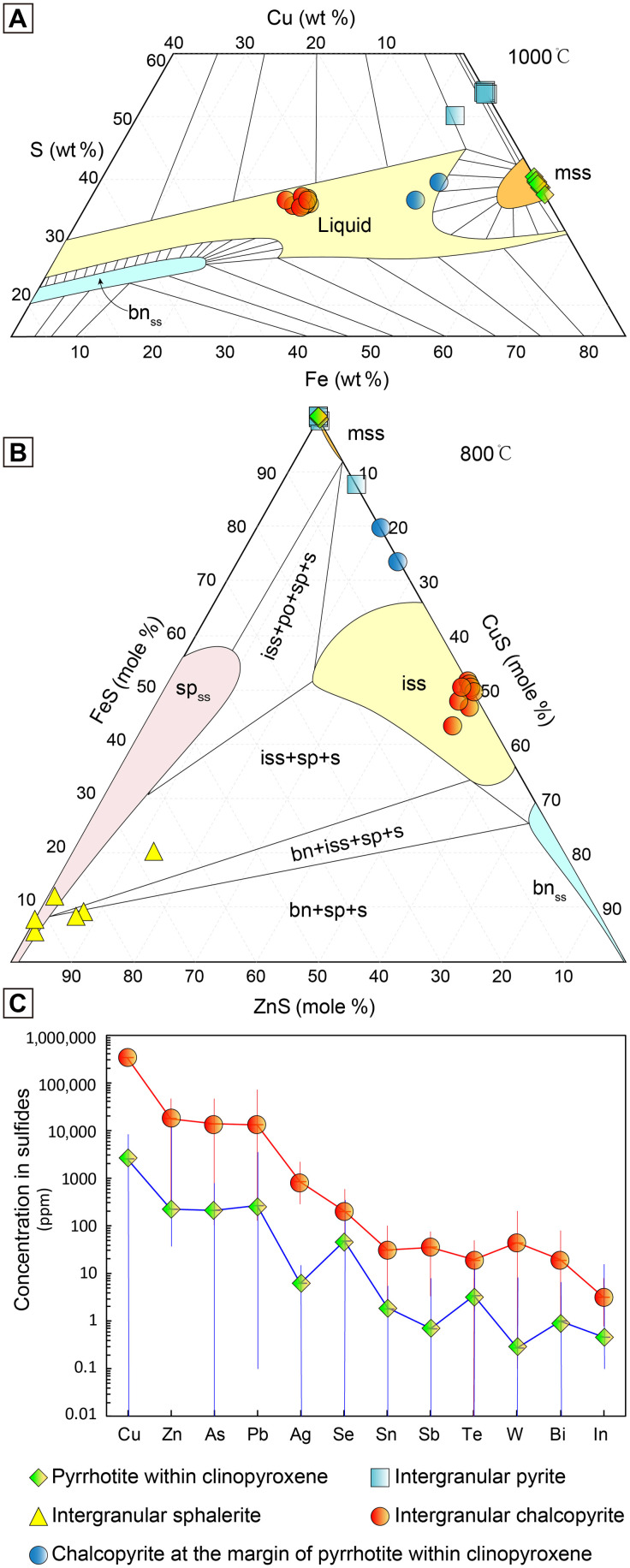
Compositional plots for sulfides from the mafic enclaves. (**A**) Compositions of sulfide inclusions projected onto the Cu-Fe-S plane and the sulfide stability fields at 1000°C and 1 atm (*27*); (**B**) sulfide inclusion compositions projected onto the CuS-FeS-ZnS plane and sulfide stability fields at 800°C and 1 bar (*29*), where bn refers to bornite; and (**C**) the chalcophile element concentrations (ranges as vertical lines) of pyrrhotite inclusions in cpx and chalcopyrite intergranular to cpx.

More than half of the analyzed pyrrhotite inclusions have the composition Fe_7_S_8_, which corresponds to that of monoclinic pyrrhotite, with a smaller subset of Fe_9_S_10_∼Fe_11_S_12_, which corresponds to hexagonal pyrrhotite (table S5). All the pyrrhotite inclusions are enriched in chalcophile metals, with average concentrations of ∼1135 ppm of Co, ∼609 ppm of Ni, ∼2628 ppm of Cu, ∼233 ppm of Zn, ∼275 ppm of Pb, ∼214 ppm of As, ∼2.0 ppm of Sn, ∼6.2 ppm of Ag, and ∼0.5 ppm of indium (In) (table S6). Chalcopyrite, which occurs at the margins of the pyrrhotite inclusions, has an elevated Fe concentration corresponding to that of intermediate solid solution (iss; Fig. 5A, ∼42 to 45 wt %) (*27*, *28*). Very small Co- and Ni-bearing grains (∼7.2% Co and ∼1.4% Ni), which are interpreted to have exsolved from the pyrrhotite, were identified in a few pyrrhotite inclusions from their energy-dispersive spectra (Fig. 3A).

Intergranular chalcopyrite contains less Fe (∼20 to 30 wt %) than chalcopyrite in the pyrrhotite globules but has a higher ZnS (zinc sulfide) concentration (up to 8 mol %; Fig. 5B) (*29*). This intergranular chalcopyrite is strongly enriched in chalcophile metals, with average concentrations of ∼1.8 wt % Zn, ∼1.37 wt % Pb, ∼1.44 wt % As, ∼30 ppm of Sn, ∼850 ppm of Ag, and ∼3.1 ppm of In (Fig. 5C). The sphalerite contains up to 20 mol % FeS [iron(II) sulfide] and 10 mol % CuS (copper sulfide) (Fig. 5B), reflecting the presence of chalcopyrite inclusions (chalcopyrite disease). This mineral was therefore not analyzed for its trace element content.

## DISCUSSION

### Sulfide saturation and the partitioning of chalcophile metals

The sulfide globules in the Beidashan mafic enclaves provide compelling evidence for the separation of a sulfide liquid from a mafic magma. The coexistence of silicate glass and pyrrhotite globules with chalcopyrite near their edges in intergranular cpx and their absence in the coarse-grained cpx indicate that a liquid, composed mainly of FeS, was the first phase to exsolve from the magma. Cpx-liquid thermobarometry and two-pyroxene thermometry show that this occurred at a temperature of 1079° to 1053°C and a pressure of ∼440 to 100 MPa, coinciding with the crystallization of the intergranular cpx in which it was trapped. As the mafic magma cooled from this temperature to below the liquidus of the sulfide melt (*30*), it crystallized monosulfide solid solution (mss), leaving a residual liquid enriched in Cu, Zn, and Pb (Fig. 3, C and F) (*27*, *31*), now preserved as inclusions in later formed hornblende and ilmenite or as intergranular sulfide melts.

The scarcity of Ni-sulfide phases and the low Ni content of the pyrrhotite (<3870 ppm) likely reflect early Ni-Co depletion (i.e., before the separation of a sulfide liquid), which we attribute to olivine (ol)/orthopyroxene fractionation into which Ni and Co would have partitioned (*D*_Ni_^ol/basalt melt^ = 24.01, *D*_Ni_^opx/basalt melt^ = 7.38, *D*_Co_^ol/basalt melt^ = 5.29, and *D*_Co_^opx/basalt melt^ = 2.48) (*32*). This mineral fractionation is consistent with the relatively high SiO_2_ contents (55.1 to 61.1 wt %) and low Ni concentrations of the mafic enclaves (*22*). Most chalcophile metals (Cu, Zn, Pb, Ag, and Sn) are incompatible in plagioclase, hornblende, and orthopyroxene, the major silicate minerals crystallizing from mafic magmas (*32*–*34*), allowing for their enrichment during olivine/orthopyroxene fractionation. However, these metals are compatible in sulfide liquids and mss, with the sulfide liquids preferring the metals over mss (*13*). Thus, the combined effects of sulfide-silicate immiscibility and continuous silicate mineral fractionation during cooling of the mafic magma led to the pronounced enrichment of many of the metals in the sulfide mineral assemblage that formed the polymetallic Weilasituo deposit with which the Beidashan pluton is associated (*D*_Cu_^ol, opx/basalt melt^ << 1, *D*_Zn_^ol, opx/basalt melt^ < 1) (*35*, *36*).

Much of the cpx, the dominant primary mafic mineral in the enclaves, was altered to hornblende (Fig. 1C). This alteration was critical for Zn enrichment, as it released Zn that had been sequestered by the cpx; whereas Zn prefers cpx over the melt, the opposite is true for hornblende (*D*_Zn_^cpx/andesite melt^ = ∼3, *D*_Zn_^hbl/andesite melt^ = ∼0.42) (*37*). The released Zn likely contributed to the Zn-rich intergranular sulfide melt and sphalerite-bearing sulfide inclusions observed in hornblende (Fig. 3E).

The solubility of sulfide in mafic silicate magmas increases strongly with decreasing pressure, and as a result, mantle-derived mafic magmas are highly undersaturated in sulfide when emplaced at shallow crustal levels (*38*). Nevertheless, sulfide saturation (due to a decrease in the sulfur-carrying capacity of silicate magmas) can occur during ascent because of the assimilation of rocks rich in silica; interaction with rocks containing reducing agents, e.g., coal measures; or interaction with rocks containing reduced sulfur (*5*, *38*, *39*). The Conrad discontinuity in the study area, which separates the felsic upper crust from the mafic lower crust, is generally present at depths of ∼24 to 38 km (corresponding to ∼670 to 1200 MPa) (*40*). Our cpx-liquid thermobarometry confirms that the first mafic-felsic magma interaction in our study area occurred under pressure-depth conditions (∼670 MPa, ∼24 km; Fig. 4B) consistent with this discontinuity, triggering the crystallization of the coarse-grained cpx. The absence of sulfide inclusions in this early-crystallized cpx, however, indicates that almost all the sulfur remained dissolved in the mafic magma until its emplacement in felsic rocks at much higher crustal levels and the crystallization of intergranular cpx (∼440 to 100 MPa, ∼16 to 3.6 km). The high Zn content of the cpx also rules out substantial sulfide segregation during magma ascent, as this would have stripped Zn from the melt. The entrapment of sulfide melt in this cpx and the coexistence of the corresponding sulfide inclusions with high-silica glass inclusions indicate that the mafic magma melted the much cooler felsic rocks and that this was the primary driver of the exsolution of a sulfide liquid from the mafic magma. This process rapidly exhausted chalcophile metals from the mafic melt (explaining the absence of sulfide inclusions in the later replacement cpx) and produced the FeS-dominated sulfide assemblage (mss + residual sulfide liquid), with high Cu, Zn, As, Pb, Ag, and Sn but low Ni and Co concentrations, in the mafic enclaves (Fig. 5C). Although Sn mineralization is typically associated with peraluminous granites and a crustal source for the Sn magmas is assumed (*10*), the elevated Sn concentrations in the immiscible sulfides in the mafic enclaves indicate that mafic magmas can also introduce significant Sn.

### Dissolution of sulfide by fluid exsolved from the mafic recharge

Bubble-bearing high-silica melt inclusions in the intergranular cpx record aqueous phase exsolution from mafic magma (Fig. 1E). This fluid release coincided with cpx replacement by hornblende and biotite, marking the onset of mafic enclave alteration by exsolved aqueous fluids documented by Raman spectroscopy in our previous study (*22*). The exclusive preservation of fine-grained pyrrhotite inclusions within pristine cpx (Fig. 3A), contrasting with the dissolution and oxidation of intergranular sulfides (Fig. 3, B to D and f), indicates that fluid exsolution closely followed sulfide melt separation. Collectively, these observations constrain a sequence of events in which the mafic magma evolved from an initial sulfide- and water-undersaturated state to near cosaturation in these phases as it was discharged into and interacted with felsic magmas in the upper crust (*22*).

The pyrrhotite inclusions in the cpx record oxygen fugacity (*f*o_2_) conditions below the pyrite-pyrrhotite buffer (approximately FMQ + 1, where FMQ is fayalite-magnetite-quartz buffer) during primary sulfide saturation of the mafic recharge system. Subsequent oxidation during the alteration of cpx to hornblende is evidenced by two key textures: (i) the replacement of pyrrhotite by pyrite along fractures in cpx (Fig. 3B) and (ii) the replacement of intergranular Cu-Fe-Zn sulfide aggregates by porous magnetite (Fig. 3F). This indicates the oxidation of sulfur from S^2−^ (pyrrhotite) to S^−1^ (pyrite) and iron from Fe^2+^ (sulfides) to Fe^3+^ (magnetite), recording a significant increase in *f*o_2_ to conditions potentially approaching those of the magnetite-hematite buffer (approximately FMQ + 2). This sulfide oxidation was followed and driven by aqueous fluid exsolution and H_2_S (hydrogen sulfide)/SO_2_ (sulfur dioxide) degassing (*15*, *41*) and potentially accompanied by ore metal mobilization.

Experimental studies have demonstrated that chalcophile metals (e.g., Cu, Zn, Pb, Ag, As, and Sn) can be transported as chloride or hydroxy complexes in aqueous fluids, with elevated chlorine (Cl) concentrations, enhancing their solubility in these fluids (*42*–*45*). Our data confirm that such fluids were present and active in metal mobilization. Electron probe microanalyzer (EPMA) analyses reveal that within individual melt inclusions, the high-silica glass proximal to vapor bubbles contains significantly higher concentrations of F (fluorine; ∼0.21 wt %), Cl (∼0.24 wt %), and SO_3_ (sulfur trioxide; ∼1.10 wt %) than glass distal from the bubbles (*22*). We interpret this compositional contrast to be the result of postentrapment diffusion from the bubbles, implying that the exsolved fluids were enriched in the ligands, particularly Cl^−^, necessary to mobilize chalcophile metals. Moreover, the preservation of partly decomposed sulfide globules (Fig. 3, B to D and F) provides unambiguous petrogenetic evidence for volatile-driven magmatic sulfide dissolution and the scavenging of chalcophile components from a precursor sulfide source (both sulfide liquid and pyrrhotite).

### Metal transfer from the mafic magma to the overlying felsic magma

The evidence presented above indicates that sulfide liquid immiscibility in the mafic magma and its dissolution by an exsolved aqueous fluid were central to metal transfer. However, once sulfide liquid exsolved from the mafic magma, it would have begun settling under gravity because of its high density relative to that of the silicate magma (5.2 to 5.8 g/cm^3^ versus 2.7 g/cm^3^ for basaltic melt). Therefore, near cosaturation and exsolution of sulfide liquid and aqueous fluid would have created a critical window for the dissolution of the sulfides by the aqueous fluid and the upward transportation of the dissolved sulfide liquid/crystals to the overlying felsic magma as discharge from the mafic recharge (*14*, *15*, *22*, *46*). In contrast, delayed fluid exsolution would have prevented the effective dissolution of the sulfide liquids/crystals in the aqueous fluid because of gravitational separation of the solute from the solvent (*47*).

**Fig. 6.**
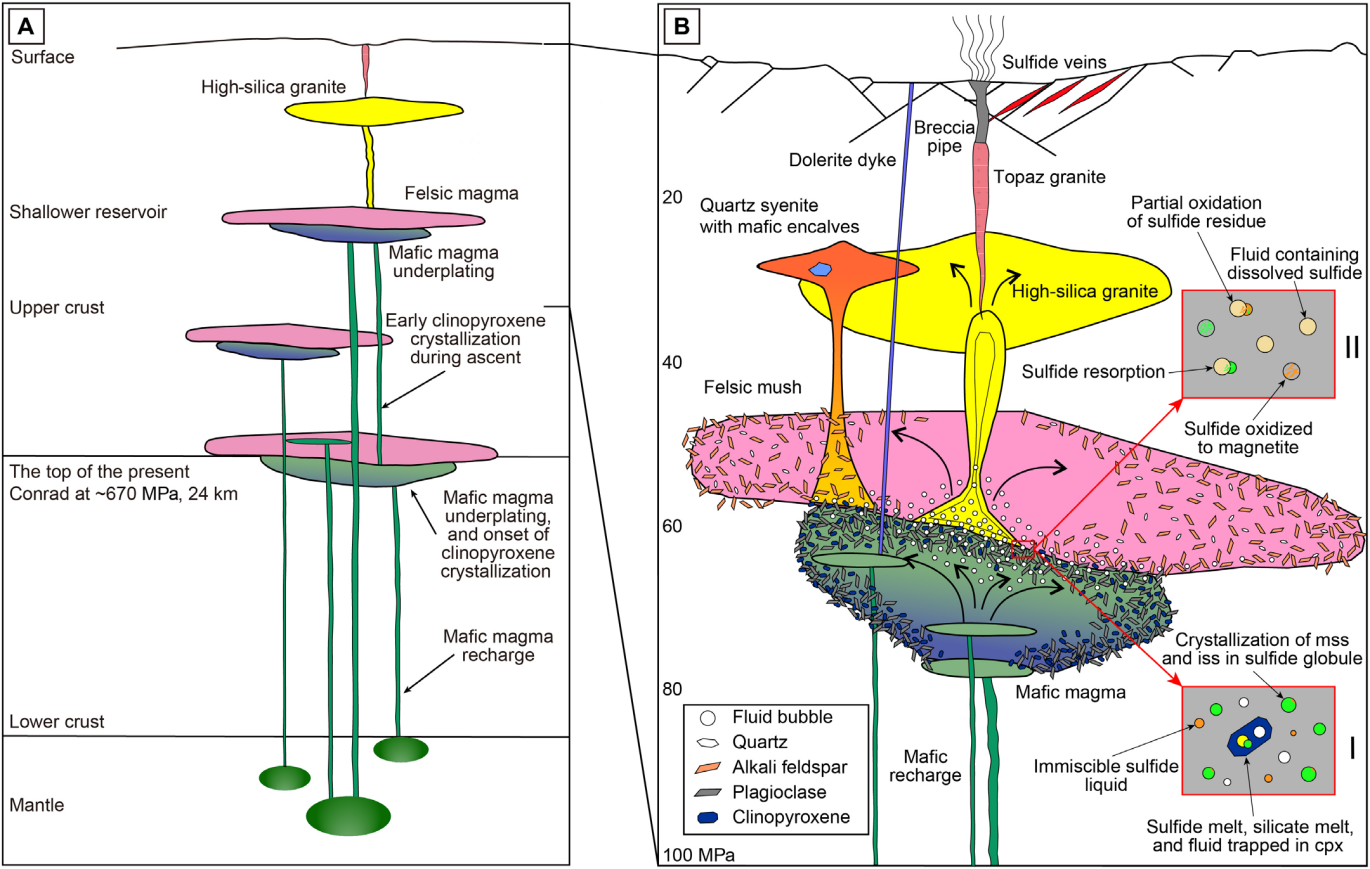
Cartoons illustrating the ascent of mantle-derived mafic magma through the crust. (**A**) Crystallization of cpx is initiated near the top of the Conrad discontinuity [the mafic-felsic interface at ∼24 km (*40*)] and continues during ascent. The upper crustal felsic magma reservoirs migrate upward as they interact with and are heated by mantle-derived mafic magma, because the expulsion of low-temperature felsic melts to shallower levels raises the effective solidus temperature of the reservoirs (*22*, *49*). (**B**) Cartoon illustrating processes in the uppermost reservoir where mafic magma reaches sulfide liquid saturation on mixing with the felsic magma (*22*). There, sulfide-silicate immiscibility is accompanied by aqueous fluid saturation, chalcophile metals are scavenged from the mafic magma by the sulfide liquid, and small samples of the sulfide liquid, high-silica felsic silicate melt, and aqueous fluid are captured by the cpx (inset image I). These sulfides and their scavenged chalcophile elements are oxidized, dissolved by the aqueous fluid (inset image II) (*14*, *15*), and lastly transported to the overlying felsic magma reservoir and site of economic mineralization.

Although contamination by external sulfur and siliceous rocks can trigger sulfide saturation (*39*), saturation of both sulfides and H_2_O in a high-temperature mafic magma can only be initiated by rapid and abundant crystallization during mafic-felsic magma interaction in response to magma mingling and underplating. This condition is consistent with the large variation in the composition of the cpx hosting pyrrhotite inclusions, from Wo_38_En_46_Fs_16_ to Wo_38_En_22_Fs_40_, which, in turn, indicates a drop in the temperature of crystallization of the intergranular cpx from ∼1079° to 1049°C (Fig. 4B). This process not only simultaneously enriched water and sulfur in the residual melt but also reduced the solubility of sulfur in the mafic melt (e.g., via FeO loss) during mafic-felsic melt interaction (*38*), thereby driving both phases to saturation at nearly the same time. Because the coalescence of sulfide melt droplets to the size necessary to settle under gravity (∼0.4 mm) only takes ∼400 years in ore-forming mafic magmas (*48*), the small pyrrhotite inclusions in the Beidashan mafic enclaves (∼10 to 25 μm) imply that sulfide saturation and H_2_O saturation were effectively coeval, assuming that the sulfur concentration was high. If, however, the sulfide abundance was low, the coalescence of exsolved sulfide liquid droplets would have been inhibited, allowing for their capture by the growing silicate crystals and inhibiting fluid resorption. Under conditions of slow fractional crystallization (without significant mafic-felsic interaction), sulfide droplets and cpx would have had time to coalesce and settle out, thereby preventing the coeval fluid-sulfide interaction required for efficient metal transfer (*48*). Therefore, only in a rapidly cooling regime of mafic recharge during mafic-felsic magma interaction can chalcophile metals be efficiently concentrated into sulfide assemblages (sulfide liquid + mss) and then dissolved in the exsolving fluid for upward discharge into the overlying felsic magma reservoir (Fig. 6) (*22*, *49*). Consequently, the relative timing of sulfide and aqueous fluid saturation in the mafic magma controls the transfer efficiency of chalcophile metals from mafic to shallow felsic reservoirs, with transfer efficiency reaching a maximum during cosaturation of water and sulfur.

A two-stage mechanism, in which chalcophile metals are first concentrated by immiscible sulfide liquids and then dissolved by coexisting aqueous fluids, constitutes a more efficient transfer pathway than direct fluid scavenging from the silicate melts. This efficiency is demonstrated in three key ways. First, the high sulfide liquid/silicate melt (sl/sm) partition coefficients [e.g., *D*_sl/sm_ ≥ 100 for Au, Cu, Ag, and Bi and *D*_sl/sm_ = 1 to 100 for Pb, Sn, Sb, and As (*12*)] enable a sulfide melt to act as a more effective initial trap than the fluid, for which the fluid/silicate melt partition coefficients are modest [e.g., *D*_fluid/sl_ = ∼1 to 20 for Zn, As, Ag, Sb, Pb, and Bi (*50*)]. Second, if sulfide liquid exsolution precedes aqueous fluid exsolution significantly, the metals would be locked into cumulates, depleting the melt and precluding effective ore formation. The coeval saturation of sulfide liquid and aqueous fluid enforced by mafic-felsic magma interaction prevents this by creating the critical window for the aqueous fluid to dissolve immiscible sulfides. Third, the model also accounts for the enrichment of Zn, which is sequestered by early-crystallizing cpx. The associated fluid-rock interaction facilitates alteration of cpx to hornblende, releasing Zn for incorporation in immiscible sulfides and their subsequent dissolution and mobilization by the fluid.

### Implications

This study provides robust evidence for the formation of felsic-associated hydrothermal sulfide deposits from chalcophile metal–rich mafic magmas. We have demonstrated that volatile-driven sulfide dissolution is the key process transferring these metals from mafic magmas to overlying felsic magmas. The process requires near-synchronous exsolution of sulfide and aqueous fluids during rapid cooling of mafic magma. Mafic-felsic magma interaction, therefore, facilitates sulfide liquid and aqueous fluid cosaturation in mafic magmas and enables efficient scavenging, dissolution, and transfer of chalcophile metals by fluid. The mafic magma source model helps explain the occurrence of chalcophile metal concentration to economic levels in felsic rock–hosted ore deposits.

## MATERIALS AND METHODS

### Analytical methods

#### Mineral and glass composition analyses

Mineral and glass (melt inclusion) compositions within cpx crystals were determined using a JXA-iHP200F EPMA equipped with five wavelength-dispersive spectrometers. The analyses were conducted at the MNR Key Laboratory of Metallogeny and Mineral Assessment, Institute of Mineral Resources, Chinese Academy of Geological Sciences (CAGS). Before analysis, thin sections were coated with a ∼20-nm-thick conductive carbon film.

The analytical procedures for silicate minerals and sulfide minerals followed (*51*), while the analysis of silicate glass (melt inclusions) within cpx followed (*52*). The specific operating conditions for silicate minerals were a 15-keV accelerating voltage, a 20-nA beam current, and a 1- to 5-μm beam diameter; for the sulfides, they were a 20-keV accelerating voltage, a 20-nA beam current, and a 1- to 5-μm beam diameter; and for the silicate glass hosted by cpx, they were a 15-keV accelerating voltage, a 10-nA beam current, and a 5- to 10-μm beam diameter. Minerals, alloys, and synthetic oxides were used as standards. The data were corrected online using a modified ZAF (atomic number, absorption, and fluorescence) correction procedure. On the basis of the counting statistics, the relative errors are estimated to be <1 to 2% at the >10 wt % level, 2 to 5% at the 10 to 3 wt % level, 5 to 10% at the 3 to 1 wt % level, and >20% at the <0.5 wt % level.

#### LA-ICP-MS in situ cpx trace element analysis

Cpx trace element analyses were conducted by laser ablation inductively coupled plasma mass spectrometry (LA-ICP-MS) at the MNR Key Laboratory of Metallogeny and Mineral Assessment, Institute of Mineral Resources, CAGS, using a RESOlution S-155 laser system coupled to a Thermo Fisher Scientific ElementXR ICP-MS. The analyses used a 35-μm-diameter laser beam operating at 6 Hz with an energy density of 5 J/cm^2^. The sample chamber was flushed with helium (0.6 liters/min), with an argon makeup gas (1.08 liters/min) introduced after the ablation. Each analysis comprised 2 s of preablation, ∼20 s of background acquisition, and ∼40 s of sample data acquisition. The NIST SRM 610 glass was used as the primary standard and the NIST SRM 612 glass as the secondary standard (*53*) in the calibration.

Given the limited variation in the SiO_2_ content of the cpx from mafic enclaves, the EPMA-determined silicon content served as the internal standard for trace element quantification. Cpx trace element concentrations were calculated using Iolite software (version 4.0) (*54*).

#### LA-ICP-MS in situ sulfide trace element analysis

Sulfide trace element analyses were conducted by LA-ICP-MS at the National Research Center for Geoanalysis, CAGS, using a New Wave NWR 193-nm ArF excimer laser system coupled to a Thermo Fisher Scientific Element2 ICP-MS. The analyses used a constant 35-μm spot size with an energy density of 3.5 J/cm^2^ at an 8-Hz repetition rate. Each spot analysis comprised 20 s of preablation, ∼20 s of background acquisition, and ∼40 s of sample data acquisition. The NIST SRM 610, KL2-G, and MASS-1 standards were used as reference materials (*53*, *55*, *56*) in the calibration, with the standards analyzed before and after every 15 sample analyses. During NIST SRM 612 line-scan ablations, the instrument’s ^232^Th sensitivity was 2 × 10^5^ cps/ppm and the ThO^+^/Th^+^ ratio < 0.3%. Element concentrations were calculated using Iolite software (*54*) with ^57^Fe as the internal standard.

Owing to the small diameter of the pyrrhotite (∼10 to 25 μm) grains, LA-ICP-MS spot analyses of pyrrhotite inclusions unavoidably ablated the surrounding cpx, producing mixed compositional data. The trace element contents of mss were therefore recalculated using the ratio of the measured Si content to the cpx Si content (measured Si/cpx Si). Similarly, ablation of chalcopyrite, which incorporates adjacent minerals, was affected by the size of the grains. On the basis of an EPMA-confirmed homogeneous Cu content in chalcopyrite (33.512 wt %), chalcopyrite trace element data were normalized using the measured Cu concentration/33.512 wt % ratio.

### *P*-*T* conditions

Cpx-liquid thermobarometry was used to estimate the crystallization temperature and pressure of cpx (*25*, *26*). For the liquid composition in these calculations, we used the most primitive sample (KLS16-2) from the mafic enclaves (*22*). The water content of the underplating mafic magma was estimated from the structurally bound water.

Cpx-liquid thermobarometry revealed a continuous *P*-*T* decrease during cpx crystallization: from high values in the coarse-grained cpx (∼670 to 320 MPa, ∼1108° to 1079°C), through lower values in the ophitic cpx with pyrrhotite (∼440 to 100 MPa, ∼1079° to 1051°C), to the significantly lower pressure and temperature recorded by ophitic overgrowth rims.

The primary uncertainty in applying cpx-liquid thermometry stems from the estimation of the liquid composition, which, because of the intense felsic-mafic interaction, was difficult to determine (*22*). The whole-rock major-element composition of sample KLS16-2 (Table 1) was used to represent the liquid composition for two key reasons: (i) It contains abundant cpx crystals with sulfide inclusions, the main target of our study, and (ii) it has the lowest SiO_2_ content (55.08 wt %) of the analyzed mafic enclaves (*22*), making it the most primitive and, thus, the best proxy for the parental melt before extensive crystallization.

**Table 1.** Major composition of the most primitive mafic enclave.

	SiO_2_	TiO_2_	Al_2_O_3_	FeO_t_	MnO	MgO	CaO	Na_2_O	K_2_O	Cr_2_O_3_	P_2_O_5_	H_2_O+
KLS16-2	55.08	1.40	17.48	7.46	0.12	2.82	7.17	3.69	2.44	0.00	0.21	1.41

**Fig. 7.**
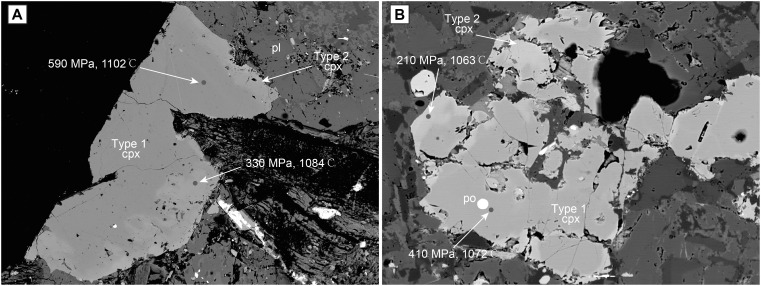
BSE images illustrating *P*-*T* differences between type 1 and type 2 cpx. (**A**) Coarse-grained cpx and (**B**) intergranular cpx with pyrrhotite inclusion.

**Fig. 8.**
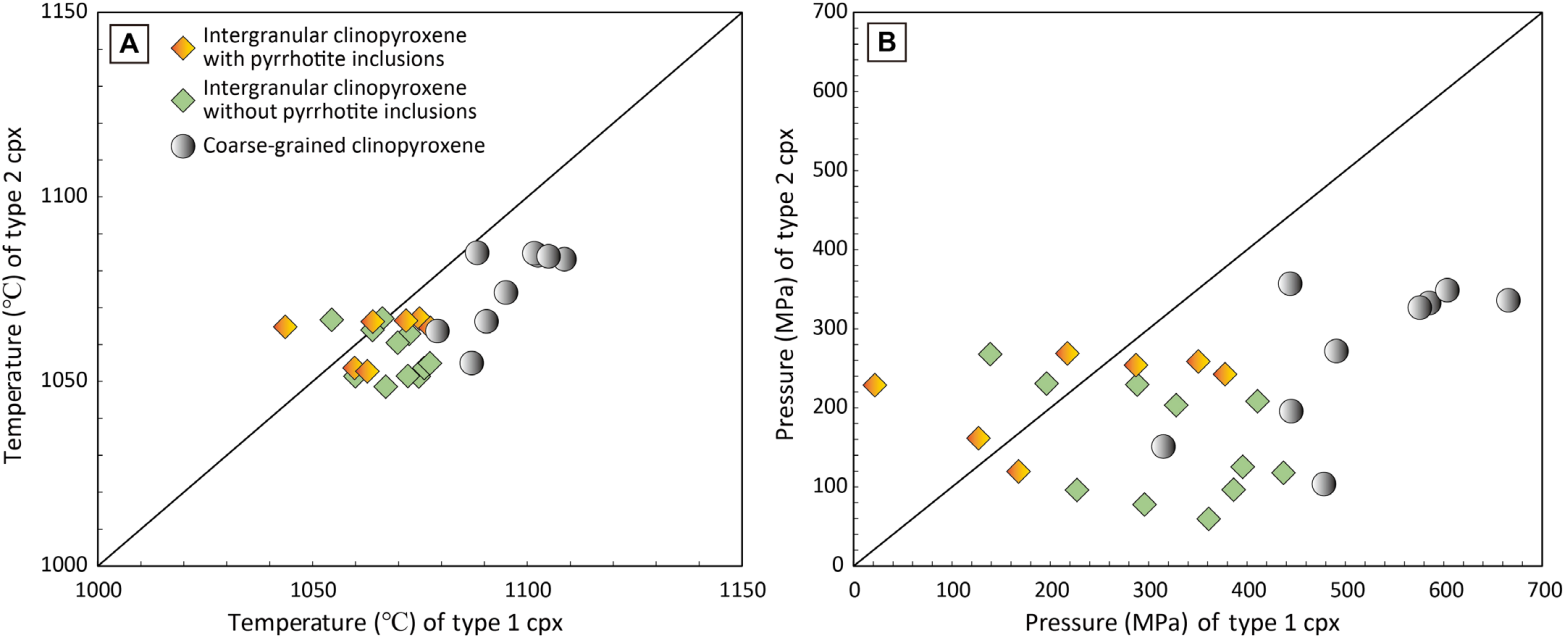
Plots of the pressure and temperature of crystallization of type 1 and type 2 cpx. (**A**) Temperature and (**B**) pressure. Most analyzed crystals record lower *P*-*T* conditions in their replaced domains (type 2 cpx).

The reliability of the cpx-liquid thermobarometry was verified by cross-checking against three independent methods:

1) Checking internal *P*-*T* consistency. A comparison of *P*-*T* conditions from cpx cores and rims shows that most crystals record significantly higher pressures in their cores (Figs. 7 and 8). This systematic core-to-rim pressure decrease is indicative of magma ascent and decompression during growth, demonstrating the internal consistency of the cpx-liquid thermobarometer.

2) Comparing with independent thermometry. There was excellent agreement between the temperatures estimated using the cpx-liquid thermobarometer and two-pyroxene thermometry (Table 2), with differences typically being within the methodological uncertainties.

**Table 2.** Comparison of cpx-liquid thermobarometers and two-pyroxene thermometers.

Clinopyroxene analysis point	Orthopyroxene analysis point	Clinopyroxene-liquid thermobarometers	Two-pyroxene thermometers	Temperature difference
KLS16-7-1.3-1	KLS16-7-1.3-2	1074°C	1058°C	16
KLS16-7-1.4-1	KLS16-7-1.4-2	1071°C	1056°C	15
KLS16-7-2.1-1	KLS16-7-2.1-2	1056°C	1012°C	44
KLS16-7-2.2-1	KLS16-7-2.2-2	1066°C	1021°C	45
KLS16-7-4.1-2	KLS16-7-4.1-3	1066°C	1020°C	46
KLS16-9-4.1	KLS16-9-4.2	1076°C	1051°C	25
KLS16-7-9.2-1	KLS16-7-9.1-1	1065°C	1018°C	47
KLS16-7-10.1-2	KLS16-7-10.1-3	1067°C	1008°C	59

**Fig. 9.**
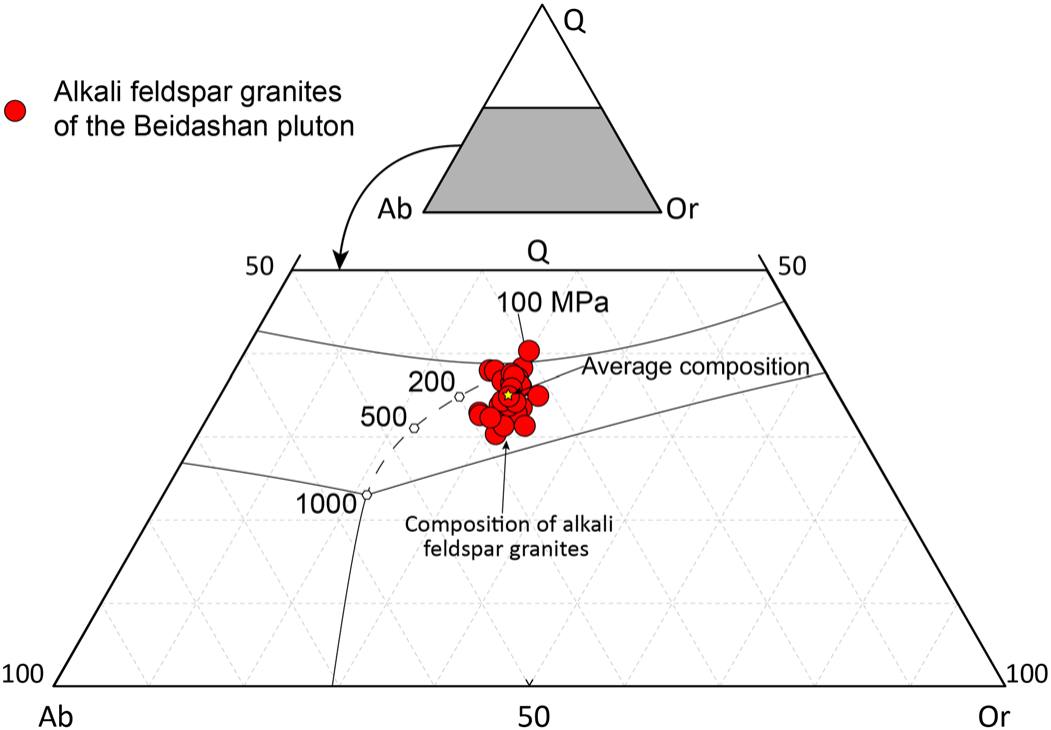
Q-Ab-Or ternary diagram for the Beidashan alkali feldspar granites. The diagram constrains the pressure of melt segregation from the magma reservoir (*22*).

3) Applying a regional granite geobarometer. The alkali feldspar granites of the Beidashan pluton, previously interpreted as melts segregated during mafic recharge and underplating (*22*), were used to provide an independent regional barometric benchmark. Application of a granite geobarometer to their major-element composition yielded pressures from >500 to <100 MPa (Fig. 9). This range closely overlaps with that obtained from cpx-liquid thermobarometry, providing strong independent support.
