# Supplementary material for: Felsic hydrothermal deposit formation by chalcophile metal concentration through mafic recharge and fluid-mediated sulfide dissolution
Source: Sci Adv. 2026 Jul 15;12(29):eaec4514. doi: 10.1126/sciadv.aec4514 (PMC13371927; doi:10.1126/sciadv.aec4514)
Supplement: Supplementary file 1 — Tables S1 to S6 Legends for data S1 and S2 [file sciadv.aec4514_sm.pdf]

Science Advances
AAAS

Supplementary Materials for

Felsic hydrothermal deposit formation by chalcophile metal concentration through mafic recharge and fluid-mediated sulfide dissolution

Yifei Liu *et al.*

Corresponding author: Yifei Liu, lyfsky@126.com

Sci. Adv. **12**, eaec4514 (2026)
DOI: 10.1126/sciadv.aec4514

The PDF file includes:

Tables S1 to S6
Legends for data S1 and S2

Other Supplementary Material for this manuscript includes the following:

Data S1 and S2

Table S1. Sample locations and descriptions used in this study

No.	Sample	Longitude	Latitude	Host lithology	Texture	Major mineralogy	Sulfide occurrence
1	KLS16-2	~E117°43.3′	~N44°05′	Quartz syenite	Fine-grained	55% pl, 15% hbl, 15% bt, 12% cpx, and 3% il	Intergranular euhedral py, euhedral py inclusions in pl and il, intergranular, anhedral ccp + sp with "ccp disease", intergranular, globular ccp bleb, globular po inclusions with expelled ccp in cpx.
2	KLS16-3	~E117°43.3′	~N44°05′	Quartz syenite	Fine-grained	55% pl, 20% cpx, 15% hbl, 12% bt, and 3% il	Intergranular euhedral py, euhedral py inclusions in pl and il, intergranular, anhedral ccp + sp with "ccp disease", globular po inclusions expelled ccp in cpx.
3	KLS16-4	~E117°43.3′	~N44°05′	Quartz syenite	Fine-grained	55% pl, 20% hbl, 15% cpx, 5% bt, and 5% il	Intergranular, tiny euhedral py, euhedral py inclusions in pl and il, intergranular, tinny, globular ccp bleb, globular po inclusions in cpx.
4	KLS16-5	~E117°43.3′	~N44°05′	Quartz syenite	Fine-grained	60% pl, 15% bt , 10% hbl, 7% cpx, and 5% il	Intergranular, tiny euhedral py, euhedral py inclusions in pl and il, tinny intergranular ccp bleb, globular po inclusions in cpx.
5	KLS16-7	~E117°43.3′	~N44°05′	Quartz syenite	Fine-grained	65% pl, 10% cpx, 5% opx,10% hbl, 5% bt, and 5% il	Intergranular, tiny euhedral py, euhedral py inclusions in pl and il, intergranular, tinny, anhedral ccp, globular po inclusions in cpx.
6	KLS16-9	~E117°43.3′	~N44°05′	Quartz syenite	Fine-grained, with coarse pl+cpx	55% pl, 20% hbl, 13% cpx, 5% bt, 5%il, and 2% opx	Intergranular, tiny, euhedral py, euhedral py inclusions in pl and il, intergranular, tinny, anhedral ccp.
7	KLS19-1	~E117°43.3′	~N44°05′	Quartz syenite	Fine-grained, with coarse pl+cpx	60% pl, 20% hbl, 10% cpx, 5% bt, and 5% il,	Intergranular, tiny, euhedral py, disseminated, tiny ccp in il, intergranular euhedral py inclusions in pl.
8	KLS19-2	~E117°43.3′	~N44°05′	Quartz syenite	Fine-grained, with coarse pl+cpx	60% pl, 20% hbl, 10% cpx, 5% bt, and 5% il,	Intergranular, tiny, euhedral py, disseminated, tiny ccp in il, intergranular euhedral py inclusions in pl.

Table S2. EPMA data (wt.%) of high-silica glassy inclusions in clinopyroxene of mafic enclaves from the Beidashan pluton (*22*)

No.	Analysis inclusion	Analysis location	SiO_2	Al_2O_3	K_2O	Na_2O	CaO	MgO	FeO	TiO_2	NiO	CuO	F	Cl	SO_3	Total
1	KLS16-2-m3	Glass on the marginal top of a nearly exposed buble	81.99	16.72	1.07	0.29	0.06	0.01	0.50	0.00	0.00	-	0.05	0.21	0.01	100.91
2	KLS16-2-m6	Glass on the central top of a nearly exposed buble	70.32	16.06	2.98	0.41	0.14	1.04	4.34	0.03	0.00	0.24	0.00	0.19	1.10	96.85
3	KLS16-2-m9-1	Galss	81.57	16.44	1.16	0.28	0.16	0.00	0.34	0.00	0.02	-	0.05	0.15	0.00	100.17
4	KLS16-2-m9-2	Glass on the central top of a nearly exposed buble	74.22	17.43	3.59	0.93	0.08	0.79	2.51	0.04	0.00	-	0.03	0.09	0.85	100.56
5	KLS16-2-m9-3	Glass on the marginal top of a nearly exposed buble	70.92	17.64	5.03	0.67	0.06	1.24	3.79	0.05	0.07	-	0.00	0.16	0.38	100.01
6	KLS16-2-m9-4	Glass on the marginal top of a nearly exposed buble	78.49	15.96	0.64	0.33	0.25	0.00	0.47	0.02	0.00	0.00	0.12	0.18	0.00	96.46
7	KLS16-2-m12	Galss	81.00	16.22	2.82	0.54	0.06	0.01	0.64	0.07	0.01	-	0.06	0.16	0.00	101.59
8	KLS16-3-m1	Galss	77.50	14.15	0.68	0.05	0.18	0.01	0.48	0.03	0.02	0.00	0.10	0.16	0.00	93.36
9	KLS16-3-m2	Galss	80.35	16.16	0.59	0.38	0.13	0.00	0.46	0.01	0.12	0.01	0.21	0.24	0.00	98.66
10	KLS16-3-m3	Galss	79.15	16.03	0.68	0.33	0.13	0.02	0.38	0.04	0.00	0.00	0.04	0.24	0.00	97.04
11	KLS19-1-m1	Galss	75.22	13.80	9.52	1.08	0.06	0.00	0.39	0.00	0.00	0.01	0.03	0.05	0.00	100.16
12	KLS19-1-m2	Glass on the central top of a nearly exposed buble	77.37	13.23	3.67	0.63	0.11	0.00	0.49	0.00	0.00	0.02	0.00	0.05	0.03	95.60
13	KLS19-1-m3	Glass on the marginal top of a nearly exposed buble	75.87	13.79	3.37	0.62	0.11	0.00	0.42	0.02	0.00	0.00	0.09	0.08	0.00	94.37
14	KLS16-9-m8	Galss	79.77	15.27	2.77	0.51	0.17	0.00	0.55	0.02	0.00	-	0.25	0.16	0.00	99.47

Table S3 EPMA data (wt.%) of pyroxenes in mafic enclaves from the Beidashan pluton and estimation of pressure-temperature conditions

No.	Analysis point	Analyzed Mineral	Sulfide inclusion, high-silica glass	Analysis site	Note
1	KLS16-2-1.1-1C	Intergranular clinopyroxene	Pyrrhotite, high-silica glass	Type 1	The same cpx grain
2	KLS16-2-1.1-2 M		-	Type 2	
3	KLS16-2-2.1C	Intergranular clinopyroxene	Pyrrhotite	Type 1	The same cpx grain
4	KLS16-2-2.1M		-	Type 2	
5	KLS16-2-3.1C	Intergranular clinopyroxene	Pyrrhotite	Type 1	The same cpx grain
6	KLS16-2-3.1M		-	Type 2	
7	KLS16-2-4.1C	Intergranular clinopyroxene	Pyrrhotite	Type 1	
8	KLS16-2-5.1C	Intergranular clinopyroxene	Pyrrhotite	Type 1	The same cpx grain
9	KLS16-2-5.1M		-	Type 2	
10	KLS16-2-6.1C	Intergranular clinopyroxene	Pyrrhotite	Type 1	The same cpx grain
11	KLS16-2-6.1M		-	Type 2	
12	KLS16-2-7.1C	Intergranular clinopyroxene	Pyrrhotite	Type 1	The same cpx grain
13	KLS16-2-7.1M		-	Type 2	
14	KLS16-2-8.1C	Intergranular clinopyroxene	Pyrrhotite	Type 1	The same cpx grain
15	KLS16-2-8.1M		-	Type 2	
16	KLS16-2-9.1C	Intergranular clinopyroxene	Pyrrhotite	Type 1	The same cpx grain
17	KLS16-2-9.1M		-	Type 2	
18	KLS16-2-10.1C	Intergranular clinopyroxene	Pyrrhotite	Type 1	The same cpx grain
19	KLS16-2-10.1M		-	Type 2	
20	KLS16-2-11.1C	Intergranular clinopyroxene	Pyrrhotite	Type 1	The same cpx grain
21	KLS16-2-11.1M		-	Type 2	
22	KLS16-2-12.1C	Intergranular clinopyroxene	Pyrrhotite	Type 1	The same cpx grain
23	KLS16-2-12.1M		-	Type 2	
24	KLS16-7-1	Intergranular orthopyroxene	-	Type 1	The same cpx grain
25	KLS16-7-1.2	Intergranular clinopyroxene	Pyrrhotite	Type 1, without Type 2	
26	KLS16-7-1.3-1	Intergranular orthopyroxene	-	Type 1, without Type 2	
27	KLS16-7-1.3-2	Intergranular clinopyroxene	Pyrrhotite	Type 1	
28	KLS16-7-1.4-1	Intergranular clinopyroxene	Pyrrhotite	Type 1, without Type 2	

No.	Analysis point	Analyzed Mineral	Sulfide inclusion, high-silica glass	Analysis site	Note
29	KLS16-7-1.4-2	Intergranular orthopyroxene	-	Type 1	
30	KLS16-7-2.1-1	Intergranular orthopyroxene	-	Type 1	
31	KLS16-7-2.1-2	Intergranular clinopyroxene	Pyrrhotite	Type 1, without Type 2	
32	KLS16-7-2.2-1	Intergranular orthopyroxene	-	Type 1	
33	KLS16-7-2.2-2	Intergranular clinopyroxene	-	Type 1, without Type 2	
34	KLS16-7-3.1	Intergranular clinopyroxene	Pyrrhotite	Type 1, without Type 2	
35	KLS16-7-4.1-1	Intergranular clinopyroxene	Pyrrhotite	Type 1, without Type 2	
36	KLS16-7-4.1-2	Intergranular orthopyroxene	-	Type 1	
37	KLS16-7-4.1-3	Intergranular clinopyroxene	-	Type 1, without Type 2	
38	KLS16-7-5.1-1	Intergranular clinopyroxene	Pyrrhotite	Type 1, without Type 2	
39	KLS16-7-5.1-2	Intergranular clinopyroxene	-	Type 2	
40	KLS16-7-5.1-3	Intergranular orthopyroxene	-	Type 1	
41	KLS16-7-6-1	Intergranular clinopyroxene	High-silica glass	Type 1	
42	KLS16-7-6-2	Intergranular clinopyroxene	-	Type 2	
43	KLS16-7-6-3	Intergranular clinopyroxene	-	Type 1, without Type 2	
44	KLS16-7-6-5	Intergranular clinopyroxene	-	Type 1, without Type 2	
45	KLS16-7-6-6	Intergranular clinopyroxene	-	Type 1, without Type 2	
46	KLS16-7-7-1	Intergranular clinopyroxene	Pyrrhotite	Type 1, without Type 2	
47	KLS16-7-8.1C	Intergranular clinopyroxene	-	Type 2	
48	KLS16-7-8.1M	Intergranular clinopyroxene	-	Type 1	
49	KLS16-7-8.1-2	Intergranular orthopyroxene	-	Type 1	
50	KLS16-7-8.1-3	Intergranular orthopyroxene	-	Type 1	
51	KLS16-7-8.1-4	Intergranular orthopyroxene	-	Type 1	
52	KLS16-7-9.2-1	Intergranular clinopyroxene	-	Type 1	The same cpx grain
53	KLS16-7-9.2-2	Intergranular clinopyroxene	-	Type 2	
54	KLS16-7-9.1-1	Intergranular orthopyroxene	-	Type 1	
55	KLS16-7-9.1-2	Intergranular orthopyroxene	-	Type 1	
56	KLS16-7-9.3-1	Intergranular clinopyroxene	-	Type 1	The same cpx grain
57	KLS16-7-9.3-2	Intergranular clinopyroxene	-	Type 2	

No.	Analysis point	Analyzed Mineral	Sulfide inclusion, high-silica glass	Analysis site	Note
58	KLS16-7-9.3-3	Intergranular clinopyroxene	-	Type 1	The same cpx grain
59	KLS16-7-9.3-4	Intergranular clinopyroxene	-	Type 2	
60	KLS16-7-9.3-5	Intergranular orthopyroxene	-	Type 1	
61	KLS16-7-10.2-1	Intergranular clinopyroxene	-	Type 1	The same cpx grain
62	KLS16-7-10.2-2	Intergranular clinopyroxene	-	Type 2	
63	KLS16-7-10.1-1	Intergranular clinopyroxene	-	Type 1	The same cpx grain
64	KLS16-7-10.1-2	Intergranular clinopyroxene	-	Type 2	
65	KLS16-7-10.1-3	Intergranular orthopyroxene	-	Type 1	
66	KLS16-4-1.1	Intergranular clinopyroxene	Pyrrhotite, high-silica glass	Type 1	
67	KLS16-4-2.1	Intergranular clinopyroxene	Pyrrhotite	Type 1	
68	KLS16-4-3.1	Intergranular clinopyroxene	Pyrrhotite	Type 1	
69	KLS16-4-4.1	Intergranular clinopyroxene	-	Type 1	
70	KLS16-4-4.2	Intergranular clinopyroxene	-	Type 1	
71	KLS16-9-1.1	Coarse clinopyroxene	Pyrrhotite	Type 1	
72	KLS16-9-2.1	Coarse clinopyroxene	Pyrrhotite	Type 1	
73	KLS16-9-3.1	Coarse clinopyroxene	Pyrrhotite	Type 1	
74	KLS16-9-4.1	Intergranular orthopyroxene	-	Type 1	
75	KLS16-9-4.2	Intergranular clinopyroxene	-	Type 1	
76	KLS16-9-5.1	Intergranular clinopyroxene	-	Type 1	
77	KLS16-9-5.2	Intergranular clinopyroxene	-	Type 1	
78	KLS16-9-6.1	Intergranular clinopyroxene	-	Type 1	
79	KLS19-1-1-1	Coarse clinopyroxene	-	Type 1	The same cpx grain
80	KLS19-1-1-2	Coarse clinopyroxene	-	Type 2	
81	KLS19-1-1-3	Coarse clinopyroxene	-	Type 1	The same cpx grain
82	KLS19-1-1-4	Coarse clinopyroxene	-	Type 2	
83	KLS19-1-1-5	Coarse clinopyroxene	-	Type 1	The same cpx grain
84	KLS19-1-1-6	Coarse clinopyroxene	-	Type 2	
85	KLS19-1-1-7	Coarse clinopyroxene	-	Type 1	The same cpx grain
86	KLS19-1-1-8	Coarse clinopyroxene	-	Type 2	

No.	Analysis point	Analyzed Mineral	Sulfide inclusion, high-silica glass	Analysis site	Note
87	KLS19-1-1-9	Coarse clinopyroxene	-	Type 1	The same cpx grain
88	KLS19-1-1-10	Coarse clinopyroxene	-	Type 2	
89	KLS19-1-2-1	Coarse clinopyroxene	-	Type 1	The same cpx grain
90	KLS19-1-2-2	Coarse clinopyroxene	-	Type 2	
91	KLS19-1-2-3	Coarse clinopyroxene	-	Type 1	The same cpx grain
92	KLS19-1-2-4	Coarse clinopyroxene	-	Type 2	
93	KLS19-1-2-5	Coarse clinopyroxene	-	Type 1	The same cpx grain
94	KLS19-1-2-6	Coarse clinopyroxene	-	Type 2	
95	KLS19-2-1.1	Coarse clinopyroxene	-	Type 1	
96	KLS19-2-2.1	Coarse clinopyroxene	-	Type 1	The same cpx grain
97	KLS19-2-2.1-1	Coarse clinopyroxene	-	Type 1	
98	KLS19-2-3.1-1	Coarse clinopyroxene	-	Type 1	The same cpx grain
99	KLS19-2-3.1-2	Coarse clinopyroxene	-	Type 2	
100	KLS19-2-3.1-3	Coarse clinopyroxene	-	Type 2 (outmost)	

Table S3 continued.

No.	Mineral major composition											
	SiO_2	TiO_2	Al_2O_3	Cr_2O_3	FeOt	MnO	MgO	CaO	Na_2O	K_2O	NiO	Total
1	52.01	0.05	0.72	0.03	15.15	0.19	11.64	19.61	0.37	0.01	0.02	99.79
2	51.36	0.06	0.46	0.00	19.84	0.11	9.31	18.30	0.24	0.02	0.01	99.70
3	52.23	0.11	0.95	0.03	13.93	0.40	12.22	19.22	0.21	0.01	0.06	99.36
4	51.74	0.12	0.39	0.00	19.01	0.25	9.18	18.73	0.26	0.08	0.03	99.78
5	52.12	0.09	0.75	0.00	14.48	0.37	11.45	19.36	0.24	0.00	0.00	98.85
6	51.70	0.08	0.41	0.00	18.26	0.22	9.54	18.71	0.19	0.02	0.01	99.12
7	52.54	0.22	1.04	0.01	13.92	0.45	12.26	19.46	0.35	0.00	0.05	100.30
8	52.40	0.15	0.94	0.00	13.52	0.42	12.32	19.51	0.33	0.00	0.01	99.59
9	51.62	0.08	0.52	0.01	19.50	0.29	9.29	18.97	0.19	0.01	0.06	100.54
10	52.57	0.12	0.75	0.00	14.23	0.34	11.61	19.85	0.37	0.00	0.00	99.85
11	52.09	0.11	0.37	0.04	17.98	0.31	10.37	19.03	0.20	0.01	0.05	100.56

No.	Mineral major composition										
	SiO_2	TiO_2	Al_2O_3	Cr_2O_3	FeOt	MnO	MgO	CaO	Na_2O	K_2O	NiO	Total
12	52.35	0.20	0.80	0.02	14.51	0.37	11.81	19.19	0.33	0.00	0.00	99.57
13	51.01	0.24	0.34	0.02	21.56	0.39	8.76	18.19	0.19	0.03	0.03	100.76
14	52.26	0.09	0.78	0.00	13.08	0.49	11.88	19.77	0.35	0.00	0.03	98.71
15	51.15	0.12	0.43	0.01	20.55	0.27	8.42	18.39	0.23	0.06	0.02	99.63
16	52.02	0.26	1.00	0.00	13.78	0.49	12.08	19.29	0.28	0.00	0.04	99.24
17	51.43	0.24	0.40	0.02	19.22	0.31	8.94	18.77	0.18	0.03	0.03	99.57
18	52.17	0.26	0.99	0.00	13.97	0.34	11.96	19.34	0.29	0.01	0.02	99.35
19	51.89	0.21	0.51	0.00	17.44	0.22	9.88	18.75	0.23	0.04	0.04	99.22
20	51.96	0.23	0.97	0.00	14.23	0.39	11.80	19.45	0.31	0.00	0.00	99.35
21	51.33	0.18	0.43	0.04	19.15	0.25	9.48	18.15	0.17	0.01	0.00	99.19
22	52.51	0.25	0.91	0.03	14.44	0.37	12.13	19.43	0.33	0.00	0.00	100.41
23	51.58	0.10	0.37	0.00	18.52	0.17	9.94	18.79	0.20	0.00	0.00	99.68
24	49.61	0.19	0.55	0.00	36.93	0.52	11.51	1.50	0.04	0.02	0.03	100.89
25	51.84	0.23	0.81	0.03	19.06	0.43	8.86	18.33	0.18	0.00	0.00	99.77
26	49.73	0.15	0.40	0.02	36.86	0.68	11.21	1.38	0.22	0.00	0.01	100.67
27	51.51	0.10	0.73	0.03	19.58	0.38	8.82	18.32	0.27	0.04	0.00	99.77
28	51.53	0.17	0.63	0.00	20.20	0.43	8.63	18.46	0.27	0.00	0.00	100.32
29	49.52	0.07	0.46	0.00	37.27	0.49	11.55	1.56	0.07	0.02	0.00	101.00
30	49.50	0.06	0.81	0.05	37.05	0.69	10.61	1.55	0.02	0.02	0.05	100.41
31	52.20	0.19	0.68	0.00	20.10	0.38	8.70	18.39	0.19	0.01	0.05	100.87
32	49.72	0.17	0.65	0.00	37.32	0.54	11.44	1.44	0.00	0.03	0.00	101.30
33	52.15	0.16	0.64	0.00	19.73	0.50	8.85	18.39	0.23	0.00	0.00	100.64
34	51.43	0.20	0.78	0.02	19.96	0.39	8.87	17.93	0.22	0.00	0.00	99.81
35	51.61	0.11	0.81	0.02	19.71	0.39	8.94	18.33	0.27	0.00	0.00	100.18
36	52.87	0.16	0.98	0.04	35.38	0.58	9.42	1.55	0.05	0.05	0.05	101.13
37	51.77	0.10	0.60	0.00	20.10	0.45	8.78	18.26	0.23	0.01	0.00	100.30
38	52.22	0.09	0.59	0.00	20.03	0.40	9.03	18.35	0.22	0.01	0.00	100.94
39	51.89	0.07	0.43	0.02	20.77	0.25	7.59	18.36	0.23	0.01	0.00	99.61

No.	Mineral major composition										
	SiO_2	TiO_2	Al_2O_3	Cr_2O_3	FeOt	MnO	MgO	CaO	Na_2O	K_2O	NiO	Total
40	49.95	0.12	0.51	0.01	36.87	0.61	11.02	1.50	0.01	0.01	0.00	100.59
41	52.25	0.17	0.39	0.00	19.95	0.44	9.21	17.85	0.19	0.00	0.04	100.49
42	51.75	0.07	0.46	0.00	20.21	0.35	7.87	19.08	0.21	0.00	0.00	100.00
43	51.77	0.11	0.32	0.00	22.02	0.60	8.50	17.19	0.16	0.00	0.00	100.65
44	51.32	0.20	0.72	0.00	19.49	0.32	8.82	18.45	0.21	0.01	0.03	99.57
45	51.87	0.15	0.33	0.02	20.15	0.38	8.39	18.37	0.22	0.00	0.00	99.87
46	51.85	0.14	0.63	0.04	19.34	0.39	9.05	18.46	0.21	0.01	0.06	100.18
47	51.59	0.78	2.19	0.01	12.22	0.35	12.81	18.95	0.29	0.00	0.00	99.18
48	51.63	0.11	0.66	0.00	19.11	0.39	9.00	18.50	0.25	0.01	0.00	99.65
49	49.86	0.14	0.58	0.02	35.73	0.70	11.19	1.45	0.00	0.01	0.00	99.68
50	49.26	0.13	0.46	0.01	36.41	0.63	11.37	1.38	0.02	0.01	0.00	99.68
51	49.95	0.17	0.69	0.00	36.08	0.41	11.04	1.55	0.02	0.01	0.00	99.92
52	51.41	0.09	0.52	0.00	19.30	0.33	8.70	18.77	0.16	0.00	0.00	99.28
53	51.50	0.07	0.23	0.00	21.32	0.33	7.48	18.13	0.23	0.00	0.00	99.29
54	49.45	0.11	0.44	0.00	37.97	0.63	10.92	1.46	0.04	0.01	0.00	101.03
55	49.15	0.10	0.42	0.00	37.97	0.79	10.53	1.42	0.04	0.00	0.04	100.47
56	51.10	0.08	0.24	0.00	22.28	0.37	7.65	17.72	0.20	0.00	0.01	99.65
57	51.25	0.08	0.57	0.00	19.27	0.36	8.48	18.55	0.19	0.01	0.00	98.75
58	51.02	0.06	0.24	0.01	21.50	0.47	7.43	18.21	0.31	0.00	0.06	99.29
59	51.56	0.06	0.61	0.04	19.26	0.37	8.66	18.57	0.24	0.00	0.00	99.36
60	48.61	0.08	1.31	0.00	36.12	0.62	10.20	1.84	0.05	0.06	0.01	98.89
61	51.25	0.04	0.25	0.00	22.32	0.42	7.19	17.89	0.25	0.01	0.00	99.62
62	51.86	0.09	0.63	0.00	19.19	0.38	8.64	18.39	0.24	0.02	0.00	99.44
63	51.39	0.05	0.24	0.00	23.06	0.37	7.08	18.38	0.23	0.01	0.00	100.81
64	52.22	0.13	0.66	0.01	19.34	0.31	8.85	18.75	0.25	0.01	0.01	100.54
65	50.04	0.09	0.68	0.01	37.01	0.66	10.86	1.55	0.05	0.03	0.00	100.97
66	52.06	0.16	0.71	0.01	19.07	0.43	8.85	18.42	0.21	0.00	0.00	99.91
67	51.71	0.20	0.79	0.00	19.33	0.46	8.68	18.43	0.26	0.01	0.02	99.88

No.	Mineral major composition											
	SiO_2	TiO_2	Al_2O_3	Cr_2O_3	FeOt	MnO	MgO	CaO	Na_2O	K_2O	NiO	Total
68	51.32	0.18	0.70	0.00	18.96	0.35	8.77	18.59	0.22	0.00	0.00	99.09
69	51.74	0.11	0.62	0.00	19.12	0.40	8.80	18.51	0.15	0.02	0.02	99.49
70	51.11	0.08	0.28	0.02	21.82	0.42	7.61	18.29	0.23	0.00	0.02	99.88
71	51.62	0.28	0.78	0.00	19.20	0.42	8.92	18.52	0.21	0.01	0.05	100.01
72	51.63	0.12	0.82	0.00	19.35	0.40	8.98	18.50	0.25	0.00	0.00	100.05
73	51.27	0.16	0.88	0.00	19.20	0.48	8.88	18.04	0.24	0.01	0.01	99.19
74	48.63	0.10	1.35	0.01	35.79	0.41	10.65	1.94	0.05	0.04	0.03	98.99
75	52.02	0.08	0.67	0.00	19.34	0.42	8.89	18.41	0.29	0.01	0.00	100.13
76	51.60	0.07	0.36	0.00	19.95	0.42	8.11	19.28	0.21	0.01	0.00	100.00
77	51.22	0.18	0.25	0.01	20.34	0.45	8.10	18.29	0.24	0.00	0.04	99.10
78	51.24	0.14	0.59	0.00	18.96	0.55	8.99	18.75	0.19	0.01	0.01	99.42
79	52.15	0.85	2.55	0.09	9.88	0.21	14.99	18.52	0.43	0.01	0.00	99.67
80	52.48	0.46	1.02	0.08	15.95	0.33	12.30	17.06	0.26	0.00	0.00	99.94
81	50.98	1.13	3.92	0.19	9.73	0.16	14.49	18.59	0.41	0.01	0.00	99.61
82	52.18	0.55	1.22	0.03	15.19	0.34	12.64	16.74	0.25	0.02	0.00	99.15
83	52.70	0.55	2.56	0.06	10.47	0.24	13.68	19.03	0.36	0.04	0.01	99.70
84	51.96	0.30	0.42	0.00	18.27	0.35	9.92	18.24	0.19	0.00	0.00	99.64
85	52.13	0.96	2.94	0.19	9.75	0.20	14.69	18.70	0.49	0.00	0.00	100.06
86	52.54	0.57	1.16	0.11	15.82	0.27	12.25	17.37	0.27	0.00	0.00	100.35
87	51.64	1.08	3.30	0.14	9.88	0.26	14.66	18.33	0.43	0.00	0.01	99.72
88	52.19	0.43	0.88	0.08	18.13	0.31	10.72	17.25	0.27	0.01	0.00	100.27
89	52.09	1.04	2.85	0.10	10.02	0.33	14.71	18.08	0.32	0.01	0.04	99.59
90	51.55	0.28	0.43	0.00	19.41	0.45	9.51	17.65	0.21	0.01	0.00	99.51
91	51.79	0.84	2.50	0.09	9.94	0.25	15.14	18.32	0.36	0.01	0.00	99.24
92	52.34	0.27	0.51	0.05	18.20	0.40	10.50	17.75	0.25	0.02	0.00	100.28
93	51.81	0.94	2.92	0.21	9.28	0.20	14.95	18.60	0.33	0.00	0.08	99.32
94	52.66	0.47	1.13	0.00	15.10	0.22	12.44	17.37	0.28	0.00	0.00	99.67
95	52.07	0.74	2.72	0.18	9.57	0.18	15.67	17.94	0.29	0.01	0.00	99.37

No.	Mineral major composition											
	SiO_2	TiO_2	Al_2O_3	Cr_2O_3	FeOt	MnO	MgO	CaO	Na_2O	K_2O	NiO	Total
96	52.01	0.75	2.65	0.17	9.31	0.16	15.78	17.92	0.37	0.00	0.00	99.12
97	52.27	0.83	1.85	0.04	11.22	0.25	14.03	18.09	0.27	0.00	0.00	98.83
98	53.10	0.85	1.69	0.07	10.43	0.26	15.23	18.09	0.27	0.00	0.00	100.00
99	52.68	0.44	0.96	0.02	16.63	0.39	11.90	17.32	0.26	0.00	0.00	100.59
100	52.01	0.25	0.49	0.02	20.75	0.29	9.09	17.61	0.20	0.01	0.02	100.74

Table S3 continued.

No.	Cation per 6 Oxygen										
	Si^{4+}	Ti^{4+}	Al^{3+}	Cr^{3+}	Fe^{2+}	Mn^{2+}	Mg^{2+}	Ca^{2+}	Na^{+}	K^{+}	Ni^{2+}
1	1.988	0.002	0.032	0.001	0.484	0.006	0.663	0.803	0.027	0.000	0.000
2	2.000	0.002	0.021	0.000	0.646	0.004	0.540	0.764	0.018	0.001	0.000
3	1.991	0.003	0.043	0.001	0.444	0.013	0.694	0.785	0.015	0.000	0.002
4	2.009	0.003	0.018	0.000	0.617	0.008	0.531	0.779	0.019	0.004	0.001
5	2.002	0.003	0.034	0.000	0.465	0.012	0.656	0.797	0.018	0.000	0.000
6	2.012	0.002	0.019	0.000	0.594	0.007	0.553	0.780	0.014	0.001	0.000
7	1.985	0.006	0.046	0.000	0.440	0.014	0.691	0.788	0.026	0.000	0.001
8	1.991	0.004	0.042	0.000	0.430	0.014	0.698	0.794	0.024	0.000	0.000
9	1.995	0.002	0.024	0.000	0.630	0.009	0.535	0.786	0.014	0.000	0.002
10	1.999	0.004	0.033	0.000	0.453	0.011	0.658	0.809	0.027	0.000	0.000
11	1.998	0.003	0.017	0.001	0.577	0.010	0.593	0.782	0.015	0.001	0.001
12	1.996	0.006	0.036	0.001	0.463	0.012	0.671	0.784	0.024	0.000	0.000
13	1.986	0.007	0.016	0.001	0.702	0.013	0.508	0.759	0.014	0.001	0.001
14	2.002	0.003	0.035	0.000	0.419	0.016	0.678	0.811	0.026	0.000	0.001
15	2.004	0.003	0.020	0.000	0.673	0.009	0.491	0.772	0.017	0.003	0.001
16	1.986	0.008	0.045	0.000	0.440	0.016	0.687	0.789	0.021	0.000	0.001
17	2.004	0.007	0.018	0.001	0.626	0.010	0.520	0.784	0.014	0.002	0.001
18	1.990	0.007	0.045	0.000	0.446	0.011	0.680	0.790	0.022	0.000	0.000
19	2.010	0.006	0.023	0.000	0.565	0.007	0.571	0.778	0.017	0.002	0.001

No.	Cation per 6 Oxygen										
	Si^{4+}	Ti^{4+}	Al^{3+}	Cr^{3+}	Fe^{2+}	Mn^{2+}	Mg^{2+}	Ca^{2+}	Na^{+}	K^{+}	Ni^{2+}
20	1.986	0.007	0.044	0.000	0.455	0.013	0.672	0.797	0.023	0.000	0.000
21	2.004	0.005	0.020	0.001	0.625	0.008	0.551	0.759	0.013	0.001	0.000
22	1.986	0.007	0.041	0.001	0.457	0.012	0.684	0.787	0.024	0.000	0.000
23	2.000	0.003	0.017	0.000	0.601	0.006	0.575	0.780	0.015	0.000	0.000
24	1.976	0.006	0.026	0.000	1.230	0.018	0.683	0.064	0.003	0.001	0.001
25	2.009	0.007	0.037	0.001	0.618	0.014	0.512	0.761	0.014	0.000	0.000
26	1.986	0.005	0.019	0.001	1.231	0.023	0.668	0.059	0.017	0.000	0.000
27	2.004	0.003	0.033	0.001	0.637	0.012	0.512	0.763	0.021	0.002	0.000
28	2.000	0.005	0.029	0.000	0.656	0.014	0.499	0.768	0.020	0.000	0.000
29	1.974	0.002	0.021	0.000	1.243	0.017	0.687	0.066	0.005	0.001	0.000
30	1.984	0.002	0.038	0.002	1.242	0.023	0.634	0.067	0.002	0.001	0.002
31	2.009	0.006	0.031	0.000	0.647	0.012	0.499	0.758	0.014	0.000	0.001
32	1.974	0.005	0.030	0.000	1.239	0.018	0.677	0.061	0.000	0.002	0.000
33	2.009	0.005	0.029	0.000	0.636	0.016	0.508	0.759	0.017	0.000	0.000
34	2.001	0.006	0.036	0.001	0.649	0.013	0.514	0.748	0.016	0.000	0.000
35	2.000	0.003	0.037	0.001	0.638	0.013	0.516	0.761	0.020	0.000	0.000
36	2.064	0.005	0.045	0.001	1.155	0.019	0.548	0.065	0.004	0.002	0.002
37	2.006	0.003	0.027	0.000	0.651	0.015	0.507	0.758	0.017	0.000	0.000
38	2.008	0.003	0.027	0.000	0.644	0.013	0.518	0.756	0.016	0.000	0.000
39	2.029	0.002	0.020	0.001	0.679	0.008	0.442	0.769	0.017	0.000	0.000
40	1.993	0.004	0.024	0.000	1.230	0.021	0.655	0.064	0.001	0.000	0.000
41	2.015	0.005	0.018	0.000	0.643	0.014	0.529	0.738	0.014	0.000	0.001
42	2.016	0.002	0.021	0.000	0.659	0.012	0.457	0.796	0.016	0.000	0.000
43	2.011	0.003	0.015	0.000	0.715	0.020	0.492	0.716	0.012	0.000	0.000
44	2.000	0.006	0.033	0.000	0.635	0.010	0.512	0.771	0.016	0.000	0.001
45	2.019	0.005	0.015	0.001	0.656	0.012	0.487	0.766	0.016	0.000	0.000
46	2.006	0.004	0.029	0.001	0.626	0.013	0.522	0.765	0.016	0.001	0.002
47	1.954	0.022	0.098	0.000	0.387	0.011	0.723	0.769	0.021	0.000	0.000

No.	Cation per 6 Oxygen										
	Si^{4+}	Ti^{4+}	Al^{3+}	Cr^{3+}	Fe^{2+}	Mn^{2+}	Mg^{2+}	Ca^{2+}	Na^{+}	K^{+}	Ni^{2+}
48	2.007	0.003	0.030	0.000	0.621	0.013	0.521	0.770	0.019	0.000	0.000
49	1.999	0.004	0.027	0.001	1.198	0.024	0.669	0.062	0.000	0.001	0.000
50	1.984	0.004	0.022	0.000	1.226	0.022	0.682	0.060	0.002	0.001	0.000
51	1.997	0.005	0.033	0.000	1.207	0.014	0.658	0.066	0.002	0.000	0.000
52	2.009	0.003	0.024	0.000	0.631	0.011	0.507	0.786	0.012	0.000	0.000
53	2.027	0.002	0.011	0.000	0.702	0.011	0.439	0.765	0.017	0.000	0.000
54	1.978	0.003	0.021	0.000	1.270	0.021	0.651	0.062	0.003	0.000	0.000
55	1.980	0.003	0.020	0.000	1.280	0.027	0.633	0.061	0.003	0.000	0.001
56	2.013	0.002	0.011	0.000	0.734	0.012	0.449	0.748	0.016	0.000	0.000
57	2.013	0.002	0.026	0.000	0.633	0.012	0.496	0.781	0.015	0.001	0.000
58	2.016	0.002	0.011	0.000	0.710	0.016	0.438	0.771	0.023	0.000	0.002
59	2.012	0.002	0.028	0.001	0.629	0.012	0.504	0.776	0.018	0.000	0.000
60	1.975	0.002	0.063	0.000	1.227	0.021	0.618	0.080	0.004	0.003	0.000
61	2.021	0.001	0.011	0.000	0.736	0.014	0.422	0.756	0.019	0.000	0.000
62	2.018	0.002	0.029	0.000	0.625	0.013	0.501	0.767	0.018	0.001	0.000
63	2.011	0.002	0.011	0.000	0.755	0.012	0.413	0.770	0.018	0.001	0.000
64	2.011	0.004	0.030	0.000	0.623	0.010	0.508	0.774	0.019	0.000	0.000
65	1.990	0.003	0.032	0.000	1.231	0.022	0.644	0.066	0.004	0.001	0.000
66	2.014	0.005	0.032	0.000	0.617	0.014	0.511	0.763	0.016	0.000	0.000
67	2.007	0.006	0.036	0.000	0.627	0.015	0.502	0.766	0.020	0.000	0.001
68	2.006	0.005	0.032	0.000	0.620	0.012	0.511	0.778	0.017	0.000	0.000
69	2.013	0.003	0.029	0.000	0.622	0.013	0.510	0.772	0.011	0.001	0.001
70	2.009	0.002	0.013	0.001	0.717	0.014	0.446	0.770	0.018	0.000	0.001
71	2.000	0.008	0.035	0.000	0.622	0.014	0.515	0.769	0.016	0.001	0.001
72	2.000	0.004	0.038	0.000	0.627	0.013	0.518	0.768	0.019	0.000	0.000
73	2.002	0.005	0.041	0.000	0.627	0.016	0.517	0.755	0.018	0.000	0.000
74	1.970	0.003	0.064	0.000	1.213	0.014	0.643	0.084	0.004	0.002	0.001
75	2.012	0.002	0.030	0.000	0.625	0.014	0.513	0.763	0.021	0.001	0.000

No.	Cation per 6 Oxygen										
	Si^{4+}	Ti^{4+}	Al^{3+}	Cr^{3+}	Fe^{2+}	Mn^{2+}	Mg^{2+}	Ca^{2+}	Na^{+}	K^{+}	Ni^{2+}
76	2.011	0.002	0.017	0.000	0.650	0.014	0.471	0.805	0.016	0.000	0.000
77	2.016	0.005	0.011	0.000	0.669	0.015	0.475	0.771	0.018	0.000	0.001
78	2.000	0.004	0.027	0.000	0.619	0.018	0.523	0.784	0.014	0.000	0.000
79	1.941	0.024	0.112	0.003	0.307	0.007	0.831	0.738	0.031	0.000	0.000
80	1.992	0.013	0.046	0.002	0.506	0.011	0.696	0.694	0.019	0.000	0.000
81	1.900	0.032	0.172	0.006	0.303	0.005	0.805	0.742	0.029	0.000	0.000
82	1.989	0.016	0.055	0.001	0.484	0.011	0.719	0.684	0.019	0.001	0.000
83	1.965	0.016	0.112	0.002	0.326	0.008	0.760	0.760	0.026	0.002	0.000
84	2.009	0.009	0.019	0.000	0.591	0.011	0.572	0.756	0.014	0.000	0.000
85	1.932	0.027	0.129	0.006	0.302	0.006	0.812	0.743	0.035	0.000	0.000
86	1.986	0.016	0.052	0.003	0.500	0.009	0.691	0.704	0.020	0.000	0.000
87	1.921	0.030	0.145	0.004	0.307	0.008	0.813	0.730	0.031	0.000	0.000
88	1.997	0.012	0.040	0.002	0.580	0.010	0.611	0.707	0.020	0.000	0.000
89	1.939	0.029	0.125	0.003	0.312	0.011	0.816	0.721	0.023	0.000	0.001
90	2.006	0.008	0.020	0.000	0.632	0.015	0.552	0.736	0.016	0.000	0.000
91	1.936	0.024	0.110	0.003	0.311	0.008	0.844	0.734	0.026	0.000	0.000
92	2.006	0.008	0.023	0.002	0.583	0.013	0.600	0.729	0.018	0.001	0.000
93	1.931	0.026	0.128	0.006	0.289	0.006	0.831	0.743	0.024	0.000	0.002
94	1.996	0.013	0.051	0.000	0.479	0.007	0.703	0.706	0.020	0.000	0.000
95	1.937	0.021	0.119	0.005	0.298	0.006	0.869	0.715	0.021	0.000	0.000
96	1.938	0.021	0.116	0.005	0.290	0.005	0.876	0.715	0.027	0.000	0.000
97	1.969	0.023	0.082	0.001	0.354	0.008	0.788	0.730	0.020	0.000	0.000
98	1.968	0.024	0.074	0.002	0.323	0.008	0.842	0.718	0.019	0.000	0.000
99	1.994	0.012	0.043	0.001	0.526	0.013	0.672	0.702	0.019	0.000	0.000
100	2.007	0.007	0.022	0.001	0.669	0.009	0.523	0.728	0.015	0.000	0.001

Table S3 continued.

No.	Cation per 6 Oxygen (after Fe^{3+} correction and site allocation)												
	Si(T)	Ti(M1)	Al^{IV}(T)	Al^{VI}(M1)	Cr(M1)	Fe^{3+}(M1)	Fe^{2+}(M1+M2)	Mn(M2)	Mg(M1+M2)	Ca(M2)	Na(M2)	K(M2)	Ni(M1)
1	1.984	0.002	0.016	0.017	0.001	0.023	0.461	0.006	0.662	0.802	0.027	0.000	0.000
2	2.002	0.002	-0.002	0.023	0.000	-0.010	0.657	0.004	0.541	0.764	0.018	0.001	0.000
3	1.995	0.003	0.005	0.038	0.001	-0.025	0.470	0.013	0.696	0.787	0.015	0.000	0.002
4	2.014	0.003	-0.014	0.032	0.000	-0.029	0.648	0.008	0.533	0.781	0.019	0.004	0.001
5	2.009	0.003	-0.009	0.043	0.000	-0.039	0.506	0.012	0.658	0.799	0.018	0.000	0.000
6	2.020	0.002	-0.020	0.039	0.000	-0.050	0.646	0.007	0.556	0.783	0.014	0.001	0.000
7	1.986	0.006	0.014	0.032	0.000	-0.006	0.446	0.014	0.691	0.788	0.026	0.000	0.001
8	1.992	0.004	0.008	0.034	0.000	-0.011	0.441	0.014	0.698	0.795	0.024	0.000	0.000
9	1.996	0.002	0.004	0.019	0.000	-0.005	0.636	0.009	0.536	0.786	0.014	0.000	0.002
10	2.002	0.004	-0.002	0.036	0.000	-0.018	0.471	0.011	0.659	0.810	0.027	0.000	0.000
11	1.999	0.003	0.001	0.016	0.001	-0.007	0.584	0.010	0.593	0.783	0.015	0.001	0.001
12	2.000	0.006	0.000	0.036	0.001	-0.024	0.488	0.012	0.672	0.786	0.024	0.000	0.000
13	1.982	0.007	0.018	-0.002	0.001	0.021	0.680	0.013	0.507	0.757	0.014	0.001	0.001
14	2.006	0.003	-0.006	0.042	0.000	-0.028	0.448	0.016	0.680	0.813	0.026	0.000	0.001
15	2.007	0.003	-0.007	0.027	0.000	-0.021	0.695	0.009	0.492	0.773	0.017	0.003	0.001
16	1.990	0.008	0.010	0.035	0.000	-0.019	0.460	0.016	0.689	0.791	0.021	0.000	0.001
17	2.011	0.007	-0.011	0.029	0.001	-0.040	0.668	0.010	0.521	0.787	0.014	0.002	0.001
18	1.994	0.007	0.006	0.039	0.000	-0.026	0.472	0.011	0.682	0.792	0.022	0.000	0.000
19	2.020	0.006	-0.020	0.043	0.000	-0.056	0.624	0.007	0.573	0.782	0.017	0.002	0.001
20	1.988	0.007	0.012	0.032	0.000	-0.010	0.465	0.013	0.673	0.797	0.023	0.000	0.000
21	2.010	0.005	-0.010	0.030	0.001	-0.038	0.665	0.008	0.553	0.761	0.013	0.001	0.000
22	1.987	0.007	0.013	0.027	0.001	-0.004	0.461	0.012	0.684	0.788	0.025	0.000	0.000
23	2.002	0.003	-0.002	0.019	0.000	-0.011	0.613	0.006	0.575	0.781	0.015	0.000	0.000
24	1.972	0.006	0.028	-0.002	0.000	0.023	1.205	0.018	0.682	0.064	0.003	0.001	0.001
25	2.023	0.007	-0.023	0.060	0.001	-0.084	0.706	0.014	0.515	0.767	0.014	0.000	0.000
26	1.982	0.005	0.018	0.001	0.001	0.024	1.204	0.023	0.666	0.059	0.017	0.000	0.000
27	2.010	0.003	-0.010	0.044	0.001	-0.038	0.677	0.012	0.513	0.766	0.021	0.002	0.000

No.	Cation per 6 Oxygen (after Fe^{3+} correction and site allocation)												
	Si(T)	Ti(M1)	Al^{IV}(T)	Al^{VI}(M1)	Cr(M1)	Fe^{3+}(M1)	Fe^{2+}(M1+M2)	Mn(M2)	Mg(M1+M2)	Ca(M2)	Na(M2)	K(M2)	Ni(M1)
28	2.005	0.005	-0.005	0.033	0.000	-0.028	0.685	0.014	0.501	0.769	0.020	0.000	0.000
29	1.966	0.002	0.034	-0.013	0.000	0.049	1.189	0.016	0.684	0.066	0.005	0.001	0.000
30	1.986	0.002	0.014	0.025	0.002	-0.013	1.256	0.023	0.635	0.067	0.002	0.001	0.002
31	2.020	0.006	-0.020	0.051	0.000	-0.068	0.719	0.012	0.502	0.763	0.014	0.000	0.001
32	1.971	0.005	0.029	0.001	0.000	0.020	1.217	0.018	0.676	0.061	0.000	0.002	0.000
33	2.019	0.005	-0.019	0.049	0.000	-0.060	0.699	0.016	0.511	0.763	0.018	0.000	0.000
34	2.009	0.006	-0.009	0.045	0.001	-0.050	0.702	0.013	0.516	0.751	0.017	0.000	0.000
35	2.005	0.003	-0.005	0.042	0.001	-0.034	0.674	0.013	0.518	0.763	0.020	0.000	0.000
36	2.111	0.005	-0.111	0.158	0.001	-0.274	1.456	0.020	0.561	0.066	0.004	0.003	0.002
37	2.013	0.003	-0.013	0.041	0.000	-0.043	0.696	0.015	0.509	0.761	0.017	0.000	0.000
38	2.016	0.003	-0.016	0.042	0.000	-0.047	0.693	0.013	0.520	0.759	0.016	0.000	0.000
39	2.045	0.002	-0.045	0.065	0.001	-0.097	0.782	0.008	0.446	0.775	0.018	0.000	0.000
40	1.997	0.004	0.003	0.021	0.000	-0.024	1.257	0.021	0.657	0.064	0.001	0.000	0.000
41	2.026	0.005	-0.026	0.044	0.000	-0.066	0.713	0.015	0.532	0.742	0.014	0.000	0.001
42	2.027	0.002	-0.027	0.048	0.000	-0.063	0.725	0.012	0.460	0.801	0.016	0.000	0.000
43	2.020	0.003	-0.020	0.034	0.000	-0.048	0.766	0.020	0.494	0.719	0.012	0.000	0.000
44	2.008	0.006	-0.008	0.041	0.000	-0.044	0.682	0.011	0.514	0.773	0.016	0.000	0.001
45	2.031	0.005	-0.031	0.046	0.001	-0.070	0.730	0.013	0.490	0.770	0.016	0.000	0.000
46	2.014	0.004	-0.014	0.043	0.001	-0.050	0.678	0.013	0.524	0.768	0.016	0.001	0.002
47	1.961	0.022	0.039	0.059	0.000	-0.042	0.431	0.011	0.726	0.772	0.022	0.000	0.000
48	2.014	0.003	-0.014	0.045	0.000	-0.047	0.670	0.013	0.523	0.773	0.019	0.000	0.000
49	2.007	0.004	-0.007	0.034	0.001	-0.049	1.252	0.024	0.672	0.062	0.000	0.001	0.000
50	1.983	0.004	0.017	0.004	0.000	0.007	1.219	0.022	0.682	0.059	0.002	0.001	0.000
51	2.006	0.005	-0.006	0.039	0.000	-0.054	1.266	0.014	0.661	0.067	0.002	0.000	0.000
52	2.018	0.003	-0.018	0.042	0.000	-0.053	0.687	0.011	0.509	0.789	0.012	0.000	0.000
53	2.040	0.002	-0.040	0.051	0.000	-0.078	0.785	0.011	0.442	0.770	0.018	0.000	0.000
54	1.973	0.003	0.027	-0.007	0.000	0.031	1.236	0.021	0.649	0.062	0.003	0.000	0.000
55	1.976	0.003	0.024	-0.004	0.000	0.026	1.251	0.027	0.631	0.061	0.003	0.000	0.001

No.	Cation per 6 Oxygen (after Fe^{3+} correction and site allocation)											
	Si(T)	Ti(M1)	Al^{IV}(T)	Al^{VI}(M1)	Cr(M1)	Fe^{3+}(M1)	Fe^{2+}(M1+M2)	Mn(M2)	Mg(M1+M2)	Ca(M2)	Na(M2)	K(M2)	Ni(M1)
56	2.020	0.002	-0.020	0.031	0.000	-0.041	0.777	0.012	0.451	0.751	0.016	0.000	0.000
57	2.024	0.002	-0.024	0.050	0.000	-0.063	0.700	0.012	0.499	0.785	0.015	0.001	0.000
58	2.022	0.002	-0.022	0.032	0.000	-0.035	0.747	0.016	0.439	0.773	0.023	0.000	0.002
59	2.021	0.002	-0.021	0.049	0.001	-0.056	0.688	0.012	0.506	0.780	0.018	0.000	0.000
60	1.978	0.002	0.022	0.041	0.000	-0.017	1.246	0.021	0.619	0.080	0.004	0.003	0.000
61	2.030	0.001	-0.030	0.042	0.000	-0.054	0.794	0.014	0.424	0.759	0.019	0.000	0.000
62	2.031	0.003	-0.031	0.060	0.000	-0.077	0.706	0.013	0.505	0.772	0.018	0.001	0.000
63	2.015	0.002	-0.015	0.026	0.000	-0.026	0.782	0.012	0.414	0.772	0.018	0.001	0.000
64	2.021	0.004	-0.021	0.051	0.000	-0.061	0.687	0.010	0.511	0.778	0.019	0.000	0.000
65	1.993	0.003	0.007	0.025	0.000	-0.019	1.252	0.022	0.645	0.066	0.004	0.001	0.000
66	2.028	0.005	-0.028	0.061	0.000	-0.083	0.704	0.014	0.514	0.769	0.016	0.000	0.000
67	2.017	0.006	-0.017	0.053	0.000	-0.061	0.692	0.015	0.505	0.770	0.020	0.000	0.001
68	2.015	0.005	-0.015	0.048	0.000	-0.057	0.679	0.012	0.513	0.782	0.017	0.000	0.000
69	2.026	0.003	-0.026	0.054	0.000	-0.074	0.700	0.013	0.514	0.777	0.011	0.001	0.001
70	2.014	0.002	-0.014	0.027	0.001	-0.028	0.747	0.014	0.447	0.772	0.018	0.000	0.001
71	2.009	0.008	-0.009	0.045	0.000	-0.054	0.679	0.014	0.518	0.772	0.016	0.001	0.001
72	2.007	0.004	-0.007	0.044	0.000	-0.039	0.668	0.013	0.520	0.770	0.019	0.000	0.000
73	2.011	0.005	-0.011	0.052	0.000	-0.054	0.685	0.016	0.520	0.758	0.018	0.000	0.000
74	1.971	0.003	0.029	0.035	0.000	-0.006	1.219	0.014	0.643	0.084	0.004	0.002	0.001
75	2.021	0.002	-0.021	0.052	0.000	-0.055	0.684	0.014	0.515	0.767	0.022	0.001	0.000
76	2.018	0.002	-0.018	0.035	0.000	-0.041	0.694	0.014	0.473	0.808	0.016	0.000	0.000
77	2.024	0.005	-0.024	0.036	0.000	-0.053	0.725	0.015	0.477	0.774	0.019	0.000	0.001
78	2.005	0.004	-0.005	0.032	0.000	-0.031	0.651	0.018	0.524	0.786	0.014	0.000	0.000
79	1.944	0.024	0.056	0.056	0.003	-0.018	0.326	0.007	0.832	0.739	0.031	0.000	0.000
80	2.002	0.013	-0.002	0.048	0.002	-0.060	0.569	0.011	0.699	0.698	0.019	0.000	0.000
81	1.903	0.032	0.097	0.075	0.006	-0.017	0.321	0.005	0.806	0.743	0.029	0.000	0.000
82	2.000	0.016	0.000	0.056	0.001	-0.069	0.556	0.011	0.723	0.687	0.019	0.001	0.000
83	1.976	0.016	0.024	0.089	0.002	-0.070	0.399	0.008	0.765	0.765	0.026	0.002	0.000

No.	Cation per 6 Oxygen (after Fe^{3+} correction and site allocation)												
	Si(T)	Ti(M1)	Al^{IV}(T)	Al^{VI}(M1)	Cr(M1)	Fe^{3+}(M1)	Fe^{2+}(M1+M2)	Mn(M2)	Mg(M1+M2)	Ca(M2)	Na(M2)	K(M2)	Ni(M1)
84	2.019	0.009	-0.019	0.038	0.000	-0.061	0.654	0.011	0.575	0.759	0.014	0.000	0.000
85	1.937	0.027	0.063	0.065	0.006	-0.026	0.329	0.006	0.813	0.744	0.035	0.000	0.000
86	1.996	0.016	0.004	0.048	0.003	-0.061	0.563	0.009	0.694	0.707	0.020	0.000	0.000
87	1.926	0.030	0.074	0.071	0.004	-0.030	0.338	0.008	0.815	0.732	0.031	0.000	0.000
88	2.007	0.012	-0.007	0.047	0.002	-0.060	0.642	0.010	0.614	0.711	0.020	0.000	0.000
89	1.948	0.029	0.052	0.074	0.003	-0.060	0.373	0.011	0.820	0.725	0.023	0.000	0.001
90	2.014	0.008	-0.014	0.034	0.000	-0.048	0.682	0.015	0.554	0.739	0.016	0.000	0.000
91	1.938	0.024	0.062	0.049	0.003	-0.011	0.322	0.008	0.845	0.735	0.026	0.000	0.000
92	2.015	0.008	-0.015	0.038	0.002	-0.050	0.636	0.013	0.602	0.732	0.018	0.001	0.000
93	1.937	0.026	0.063	0.066	0.006	-0.038	0.328	0.006	0.833	0.745	0.024	0.000	0.002
94	2.009	0.014	-0.009	0.060	0.000	-0.076	0.557	0.007	0.707	0.710	0.020	0.000	0.000
95	1.941	0.021	0.059	0.061	0.005	-0.028	0.327	0.006	0.871	0.717	0.021	0.000	0.000
96	1.941	0.021	0.059	0.057	0.005	-0.018	0.308	0.005	0.878	0.716	0.027	0.000	0.000
97	1.981	0.024	0.019	0.064	0.001	-0.074	0.429	0.008	0.793	0.735	0.020	0.000	0.000
98	1.979	0.024	0.021	0.053	0.002	-0.062	0.387	0.008	0.846	0.722	0.019	0.000	0.000
99	2.003	0.013	-0.003	0.046	0.001	-0.056	0.585	0.013	0.675	0.706	0.019	0.000	0.000
100	2.016	0.007	-0.016	0.038	0.001	-0.054	0.726	0.009	0.525	0.731	0.015	0.000	0.001

Table S3 continued.

No.	Mg#	Wo	En	Fs	Clinopyroxene-based Thermobarometers		Two pyroxene Thermometers
					T(C) Eqn. 33, Putirka, K. (2008)	P(kbar) N&P 2017	T(C) Eqn. 36, Putirka, K. (2008)
1	0.59	0.42	0.34	0.24	1066	2.88	
2	0.45	0.39	0.28	0.33	1067	2.29	
3	0.60	0.40	0.36	0.24	1054	1.39	
4	0.45	0.40	0.27	0.33	1067	2.68	
5	0.57	0.41	0.34	0.26	1060	2.27	
6	0.46	0.39	0.28	0.33	1051	0.96	
7	0.61	0.41	0.36	0.23	1079	4.22	

No.	Mg#	Wo	En	Fs	Clinopyroxene-based Thermobarometers		Two pyroxene Thermometers
					T(C) Eqn. 33, Putirka, K. (2008)	P(kbar) N&P 2017	T(C) Eqn. 36, Putirka, K. (2008)
8	0.61	0.41	0.36	0.23	1075	3.86	
9	0.46	0.40	0.27	0.32	1051	0.96	
10	0.58	0.42	0.34	0.24	1076	4.37	
11	0.50	0.40	0.30	0.30	1054	1.18	
12	0.58	0.40	0.35	0.25	1076	3.88	
13	0.43	0.39	0.26	0.35	~~958~~	~~-10.11~~	
14	0.60	0.42	0.35	0.23	1072	4.11	
15	0.41	0.39	0.25	0.35	1063	2.08	
16	0.60	0.41	0.36	0.24	1067	2.96	
17	0.44	0.40	0.26	0.34	1049	0.78	
18	0.59	0.41	0.35	0.24	1070	3.28	
19	0.48	0.40	0.29	0.32	1061	2.04	
20	0.59	0.41	0.35	0.24	1072	3.61	
21	0.45	0.38	0.28	0.34	1051	0.60	
22	0.60	0.41	0.35	0.24	1077	3.96	
23	0.48	0.40	0.29	0.31	1055	1.25	
24	0.36	0.03	0.35	0.62			
25	0.42	0.39	0.26	0.36	1053	1.00	
26	0.36	0.03	0.35	0.62			1058
27	0.43	0.39	0.26	0.35	1074	3.19	
28	0.42	0.39	0.26	0.35	1071	2.94	1056
29	0.37	0.03	0.35	0.61			
30	0.34	0.03	0.32	0.64			1012.3
31	0.41	0.38	0.25	0.36	1056	1.16	
32	0.36	0.03	0.35	0.62			1020.7
33	0.42	0.39	0.26	0.35	1066	2.33	
34	0.42	0.38	0.26	0.36	1066	2.07	
35	0.43	0.39	0.26	0.34	1074	3.11	

No.	Mg#	Wo	En	Fs	Clinopyroxene-based Thermobarometers		Two pyroxene Thermometers
					T(C) Eqn. 33, Putirka, K. (2008)	P(kbar) N&P 2017	T(C) Eqn. 36, Putirka, K. (2008)
36	0.28	0.03	0.27	0.70			1020.4
37	0.42	0.39	0.26	0.35	1066	2.20	
38	0.43	0.38	0.26	0.35	1064	1.96	
39	0.36	0.39	0.22	0.39	1064	2.31	
40	0.34	0.03	0.33	0.64			
41	0.43	0.37	0.27	0.36	1060	1.27	
42	0.39	0.40	0.23	0.37	1054	1.62	
43	0.39	0.36	0.25	0.39	1056	0.45	
44	0.43	0.39	0.26	0.35	1059	1.73	
45	0.40	0.39	0.25	0.37	1061	1.88	
46	0.44	0.39	0.27	0.34	1060	1.73	
47	0.63	0.40	0.38	0.22	1075	3.50	
48	0.44	0.39	0.27	0.34	1067	2.59	
49	0.35	0.03	0.34	0.63			
50	0.36	0.03	0.35	0.62			
51	0.34	0.03	0.33	0.63			
52	0.43	0.40	0.26	0.35	1044	0.22	
53	0.36	0.39	0.22	0.39	1065	2.29	1017.7
54	0.34	0.03	0.33	0.63			
55	0.34	0.03	0.32	0.64			
56	0.37	0.38	0.23	0.39	1063	1.68	
57	0.42	0.40	0.25	0.35	1053	1.20	
58	0.37	0.39	0.22	0.38	1077	3.78	
59	0.42	0.40	0.26	0.35	1065	2.43	
60	0.33	0.04	0.32	0.64			
61	0.35	0.38	0.21	0.40	1072	2.87	
62	0.42	0.39	0.25	0.36	1067	2.54	
63	0.35	0.39	0.21	0.40	1064	2.17	

No.	Mg#	Wo	En	Fs	Clinopyroxene-based Thermobarometers		Two pyroxene Thermometers
					T(C) Eqn. 33, Putirka, K. (2008)	P(kbar) N&P 2017	T(C) Eqn. 36, Putirka, K. (2008)
64	0.43	0.39	0.26	0.35	1067	2.69	1007.8
65	0.34	0.03	0.33	0.64			
66	0.42	0.39	0.26	0.35	1060	1.78	
67	0.42	0.39	0.26	0.35	1070	2.94	
68	0.43	0.40	0.26	0.34	1060	2.04	
69	0.42	0.39	0.26	0.35	~~1043~~	~~-0.04~~	
70	0.37	0.39	0.23	0.38	1064	2.20	
71	0.43	0.39	0.26	0.34	1059	1.69	
72	0.44	0.39	0.27	0.34	1069	2.75	
73	0.43	0.39	0.26	0.35	1070	2.67	
74	0.35	0.04	0.33	0.63			1050.7
75	0.43	0.39	0.26	0.35	1076	3.45	
76	0.41	0.41	0.24	0.35	1050	1.33	
77	0.40	0.39	0.24	0.37	1065	2.48	
78	0.45	0.40	0.27	0.33	1050	0.95	
79	0.72	0.39	0.44	0.17	1102	5.85	
80	0.55	0.35	0.36	0.29	1084	3.33	
81	0.72	0.40	0.43	0.17	1102	5.76	
82	0.57	0.35	0.37	0.28	1085	3.27	
83	0.66	0.40	0.40	0.21	1087	4.78	
84	0.47	0.38	0.29	0.33	1055	1.04	
85	0.71	0.39	0.43	0.17	1109	6.66	
86	0.55	0.36	0.35	0.29	1083	3.36	
87	0.71	0.39	0.43	0.18	1105	6.04	
88	0.49	0.36	0.31	0.33	1084	3.49	
89	0.69	0.38	0.43	0.19	1090	4.45	
90	0.45	0.37	0.28	0.35	1066	1.96	
91	0.72	0.39	0.44	0.17	1095	4.91	

No.	Mg#	Wo	En	Fs	Clinopyroxene-based Thermobarometers		Two pyroxene Thermometers
					T(C) Eqn. 33, Putirka, K. (2008)	P(kbar) N&P 2017	T(C) Eqn. 36, Putirka, K. (2008)
92	0.49	0.37	0.31	0.32	1074	2.72	
93	0.72	0.39	0.44	0.17	1088	4.44	
94	0.56	0.36	0.36	0.28	1085	3.57	
95	0.73	0.37	0.45	0.17	1087	3.77	
96	0.74	0.38	0.46	0.16	1101	5.31	
97	0.65	0.38	0.41	0.22	1079	3.34	
98	0.69	0.37	0.43	0.20	1079	3.15	
99	0.54	0.36	0.34	0.30	1082	3.15	
100	0.42	0.37	0.26	0.37	1064	1.51	

Table S4. LA-ICP-MS trace elements concentration (ppm) of clinopyroxene with sulfide inclusions in mafic enclaves

Sample		KLS19-1- 1	KLS19-1- 2	KLS19-1- 3	KLS19-1- 4	KLS19-1- 6	KLS19-1- 7	KLS19-1- 8	KLS19-1- 9	Average
Si	wt%	24.23	24.23	24.23	24.23	24.23	24.23	24.23	24.23	
Na	ppm	3104	2327	2055	2590	2886	2190	4395	2387	2742
Mg	ppm	76849	74007	71966	73575	73912	74974	74456	75814	74444
Mg	ppm	81913	80396	76842	78676	78122	79753	80107	80410	79527
Al	ppm	18125	16966	17377	16879	17398	15878	17756	16860	17155
P	ppm	90	67	67	59	58	66	53	63	65
S	ppm	364	482	470	474	313	445	581	440	446
Cl	ppm	260	302	365	328	327	346	277	312	315
K	ppm	0	20	50	17	22	0	639	0	94
Ca	ppm	122658	127073	129216	133048	128115	135918	133034	136550	130701
Sc	ppm	81	85	83	82	85	92	83	87	85
Ti	ppm	5873	5885	5794	5845	6265	5682	5480	5890	5839
Ti	ppm	5858	5861	5885	5843	6242	5685	5488	6018	5860
V	ppm	395	446	441	418	430	471	411	432	431
Cr	ppm	1004	741	702	1183	1252	459	726	1169	904
Mn	ppm	1651	1768	1855	1598	1729	1770	1702	1705	1722
Fe	ppm	72766	77615	79382	70656	73306	76840	75361	73961	74986
Co	ppm	39	39	38	38	40	40	42	38	39
Ni	ppm	19.6	17.6	15.6	19.5	16.9	14.7	19.2	17.8	17.6
Cu	ppm	0.0	0.0	0.9	0.5	0.8	0.0	0.9	0.8	0.5
Zn	ppm	102.4	114.9	117.4	100.3	103.5	102.4	94.7	103.0	104.8
Ga	ppm	11.2	11.3	11.3	10.2	11.1	11.1	11.3	10.7	11.0
Ge	ppm	9.7	12.2	9.9	10.7	11.1	12.2	10.6	12.8	11.2
As	ppm	1.6	1.6	3.0	2.8	1.9	1.8	1.5	2.3	2.1
Se	ppm	0.0	1.8	0.0	2.3	0.0	2.4	2.1	2.6	1.4
Rb	ppm	0.0	0.0	0.0	0.0	0.0	0.0	0.0	0.0	0.0
Sr	ppm	57.7	53.4	56.9	60.7	53.2	53.8	64.6	62.4	57.8
Y	ppm	21.5	22.0	26.4	21.8	22.6	24.7	22.2	23.6	23.1

Sample		KLS19-1- 1	KLS19-1- 2	KLS19-1- 3	KLS19-1- 4	KLS19-1- 6	KLS19-1- 7	KLS19-1- 8	KLS19-1- 9	Average
Zr	ppm	54.9	54.2	58.9	51.4	54.9	56.8	55.1	57.2	55.4
Nb	ppm	0.1	0.0	0.1	0.2	0.0	0.1	0.0	0.3	0.1
Mo	ppm	0.3	0.1	0.2	0.0	0.0	0.0	0.1	0.0	0.1
Rh	ppm	0.0	0.0	0.0	0.0	0.0	0.0	0.0	0.0	0.0
Pd	ppm	0.0	0.0	0.0	0.0	0.1	0.0	0.0	0.0	0.0
Ag	ppm	0.0	0.0	0.0	0.0	0.0	0.0	0.0	0.0	0.0
Cd	ppm	0.0	0.0	0.0	0.0	0.0	0.0	0.0	0.0	0.0
In	ppm	0.0	0.1	0.1	0.0	0.1	0.1	0.0	0.1	0.1
Sn	ppm	0.0	0.0	0.0	0.0	0.0	0.0	0.0	0.0	0.0
Sb	ppm	0.0	0.0	0.6	0.0	0.0	0.0	0.0	0.0	0.1
Te	ppm	0.0	0.0	0.0	0.0	0.0	0.0	0.0	0.0	0.0
Cs	ppm	0.0	0.0	0.2	0.0	0.0	0.0	0.1	0.0	0.0
Ba	ppm	1.3	0.0	0.0	0.0	0.0	0.0	25.0	0.0	3.3
La	ppm	3.6	4.1	5.8	3.8	4.0	4.4	4.6	5.3	4.4
Ce	ppm	16.7	18.0	23.1	16.5	16.3	18.8	17.2	20.4	18.4
Pr	ppm	3.6	3.5	4.4	3.4	3.4	3.7	3.7	3.9	3.7
Nd	ppm	22.0	20.2	24.9	18.5	20.4	23.0	18.7	23.9	21.5
Nd	ppm	19.6	18.9	23.2	19.4	20.0	20.4	19.1	22.9	20.4
Sm	ppm	5.7	6.7	6.3	6.6	6.3	8.4	6.7	6.7	6.7
Eu	ppm	1.8	1.7	1.5	1.4	1.7	1.5	2.0	2.0	1.7
Eu	ppm	1.6	1.5	1.5	1.4	1.8	1.9	1.7	2.0	1.7
Gd	ppm	6.4	5.9	7.5	6.0	5.5	7.4	6.7	7.0	6.6
Gd	ppm	5.9	7.5	6.6	6.4	6.5	7.2	5.5	7.6	6.7
Tb	ppm	0.9	0.7	1.1	1.0	0.8	1.1	0.9	0.9	0.9
Dy	ppm	4.9	5.3	5.9	4.9	5.5	5.5	6.1	5.7	5.5
Er	ppm	2.1	2.0	2.7	2.3	2.2	2.8	2.2	2.1	2.3
Tm	ppm	0.3	0.3	0.3	0.4	0.3	0.3	0.3	0.2	0.3
Yb	ppm	1.5	2.0	2.2	1.4	1.1	1.4	1.9	2.3	1.7
Lu	ppm	0.3	0.2	0.3	0.2	0.2	0.3	0.3	0.2	0.3

Sample		KLS19-1- 1	KLS19-1- 2	KLS19-1- 3	KLS19-1- 4	KLS19-1- 6	KLS19-1- 7	KLS19-1- 8	KLS19-1- 9	Average
Hf	ppm	2.7	2.4	2.1	2.0	2.4	1.9	2.5	1.5	2.2
Ta	ppm	0.0	0.0	0.0	0.0	0.0	0.0	0.0	0.0	0.0
W	ppm	0.0	0.0	0.0	0.0	0.0	0.0	0.0	0.0	0.0
Re	ppm	0.0	0.0	0.0	0.0	0.0	0.0	0.0	0.0	0.0
Pt	ppm	0.0	0.0	0.0	0.2	0.0	0.0	0.0	0.0	0.0
Au	ppm	0.0	0.0	0.0	0.0	0.0	0.0	0.0	0.0	0.0
Tl	ppm	0.0	0.0	0.0	0.0	0.0	0.0	0.0	0.0	0.0
Pb	ppm	0.2	0.3	0.4	0.2	0.8	0.1	0.6	0.0	0.3
Bi	ppm	0.0	0.0	0.0	0.1	0.0	0.0	0.0	0.0	0.0
Th	ppm	0.0	0.1	0.2	0.1	0.0	0.1	0.1	0.1	0.1
U	ppm	0.0	0.0	0.1	0.0	0.0	0.0	0.0	0.1	0.0

Table S4 continued.

External Standards		External Standard G_NIST610										External Standard G_NIST612				
		1	2	3	4	5	6	7	8	9	10	1	2	3	4	5
Si	wt%															
Na	ppm	99181	99829	98842	99824	97822	100965	99825	99983	99230	99209	96623	95272	94515	93006	97260
Mg	ppm	436	431	430	434	429	439	437	434	432	431	74	72	73	70	74
Mg	ppm	446	425	431	417	428	437	436	437	422	435	50	53	57	60	60
Al	ppm	10783	10826	10834	10748	10601	10983	10858	10820	10696	10862	11386	11274	11122	11163	11192
P	ppm	408	414	405	425	409	434	404	413	415	413	43	50	42	42	43
S	ppm	555	636	581	399	648	523	592	580	531	615	328	390	0	431	0
Cl	ppm	280	269	302	237	255	278	270	305	246	281	347	304	246	361	519
K	ppm	463	462	457	468	463	476	454	471	459	466	57	73	64	78	67
Ca	ppm	82550	81032	81732	82845	81408	83828	82338	82020	81983	82222	80489	78012	80005	76837	77057
Sc	ppm	457	455	453	459	451	454	463	456	452	455	38	38	38	39	37
Ti	ppm	457	449	457	446	453	447	456	460	457	442	39	34	33	36	41
Ti	ppm	454	459	446	453	447	449	455	455	447	457	39	40	37	44	43
V	ppm	450	451	451	451	443	454	445	457	451	448	40	38	39	38	39

External Standards		External Standard G_NIST610										External Standard G_NIST612				
		1	2	3	4	5	6	7	8	9	10	1	2	3	4	5
Cr	ppm	415	404	413	400	386	418	416	407	404	407	44	47	38	46	34
Mn	ppm	448	443	440	446	441	444	442	444	443	449	39	37	37	37	37
Fe	ppm	489	442	488	435	447	440	442	501	473	448	39	0	0	0	67
Co	ppm	405	415	409	412	403	411	408	412	408	412	36	34	35	35	37
Ni	ppm	461.8	460.2	452.9	469.1	446.3	457.7	463.8	461.8	462.0	458.5	43.6	37.0	37.9	37.5	40.1
Cu	ppm	441.4	445.7	441.3	440.1	433.4	434.3	445.0	446.1	432.3	449.8	40.1	36.0	33.3	37.0	32.6
Zn	ppm	459.2	449.6	472.6	460.1	464.9	459.5	452.6	456.4	461.3	459.9	38.1	39.8	42.3	38.6	39.6
Ga	ppm	436.0	436.3	428.4	429.9	430.0	434.3	436.7	436.3	420.3	441.0	37.3	32.9	38.4	37.2	36.5
Ge	ppm	455.6	440.2	441.5	443.8	445.1	452.2	438.8	456.7	446.6	448.9	40.3	40.4	44.7	36.7	39.4
As	ppm	324.0	328.4	320.2	328.0	316.5	328.6	320.1	330.7	322.3	330.0	34.8	32.9	33.3	32.3	32.0
Se	ppm	138.5	134.7	139.6	139.2	138.8	137.5	139.4	139.1	134.1	137.8	11.7	13.0	11.3	13.1	12.2
Rb	ppm	418.9	436.8	429.8	428.6	419.7	427.1	420.7	436.4	423.0	428.6	32.0	30.6	30.6	30.0	32.0
Sr	ppm	515.8	513.5	519.7	517.5	506.5	514.3	514.5	522.4	511.5	521.3	77.1	77.9	76.7	76.8	77.3
Y	ppm	453.9	471.8	465.4	458.3	459.0	458.2	460.7	472.7	459.2	462.3	38.7	37.3	37.4	38.1	38.2
Zr	ppm	436.2	458.2	453.9	448.0	442.4	448.8	437.1	456.2	447.5	452.4	39.0	37.4	37.2	39.6	39.2
Nb	ppm	455.4	477.9	467.8	464.7	462.4	472.3	456.9	481.6	463.9	465.6	38.8	38.3	38.3	36.9	37.2
Mo	ppm	411.7	417.1	419.0	423.1	417.0	418.2	406.5	425.1	418.1	418.8	37.3	35.9	38.1	35.7	36.0
Rh	ppm	1.2	1.3	1.3	1.3	1.3	1.6	1.2	1.3	1.2	1.5	0.9	0.9	0.9	0.8	0.8
Pd	ppm	0.0	1.7	1.3	1.1	1.1	1.4	1.4	1.4	0.9	1.3	1.1	0.0	0.0	0.9	0.0
Ag	ppm	245.8	256.3	251.3	250.5	246.8	248.8	252.6	260.9	249.8	248.0	22.1	21.1	21.1	23.0	21.3
Cd	ppm	278.7	267.6	262.7	273.8	272.7	257.8	270.6	290.3	269.0	268.5	28.1	25.0	28.1	26.8	27.2
In	ppm	430.1	434.6	432.6	433.0	443.5	439.0	434.2	437.9	425.4	436.9	36.4	35.2	34.8	36.2	36.6
Sn	ppm	428.5	436.0	434.0	428.0	425.2	427.2	428.6	438.4	427.5	429.9	37.5	38.8	37.7	38.9	36.1
Sb	ppm	400.9	392.2	408.7	391.5	399.1	388.3	394.9	408.7	395.6	390.1	35.0	35.0	33.0	32.0	32.5
Te	ppm	301.6	311.0	293.2	302.9	307.5	296.0	289.7	299.7	309.3	302.5	32.9	35.9	34.9	30.3	33.7
Cs	ppm	361.8	374.7	363.4	369.6	369.2	371.2	366.2	374.5	361.4	365.6	41.9	41.0	39.6	41.9	42.0
Ba	ppm	460.2	459.6	453.3	443.3	440.2	446.7	457.9	459.5	462.4	446.5	44.4	37.1	39.6	35.6	39.5
La	ppm	434.4	446.3	446.1	442.6	436.2	442.4	440.0	445.5	435.0	440.0	36.6	37.1	35.6	35.7	35.2

External Standards		External Standard G_NIST610										External Standard G_NIST612				
		1	2	3	4	5	6	7	8	9	10	1	2	3	4	5
Ce	ppm	452.2	454.0	450.6	456.7	445.3	457.1	448.5	460.8	451.7	454.3	38.2	39.0	38.3	37.7	37.8
Pr	ppm	448.0	446.6	448.9	448.4	444.0	454.1	446.0	452.4	445.4	449.3	37.6	38.2	38.3	36.8	37.6
Nd	ppm	432.1	427.3	431.7	429.4	421.8	440.3	424.5	436.7	429.1	437.8	37.5	33.2	33.8	36.8	36.7
Nd	ppm	426.7	430.5	438.5	426.6	428.6	434.4	422.5	440.7	432.4	426.2	34.2	40.1	33.7	35.3	35.2
Sm	ppm	461.1	452.9	445.6	449.1	454.4	455.9	462.2	464.2	448.2	451.7	42.5	39.0	37.2	37.2	37.3
Eu	ppm	450.2	449.3	443.4	445.7	440.5	453.7	442.9	454.7	443.2	450.7	34.1	35.4	34.3	34.2	35.9
Eu	ppm	449.8	443.6	446.9	447.5	452.6	450.4	442.5	456.6	436.3	458.9	36.8	35.0	38.4	34.0	36.4
Gd	ppm	436.7	453.7	455.8	458.0	455.2	447.7	437.9	453.2	446.7	458.2	39.3	32.6	36.4	35.6	37.7
Gd	ppm	446.9	457.8	453.0	449.4	436.2	447.6	440.0	452.8	452.0	455.4	37.3	35.7	37.1	36.6	39.9
Tb	ppm	436.5	443.5	439.2	432.6	429.7	440.0	435.0	442.0	434.3	443.4	36.8	36.6	35.7	35.7	36.0
Dy	ppm	441.8	439.2	429.6	437.2	435.8	429.6	437.9	441.4	434.7	446.2	35.6	36.0	35.6	33.5	36.9
Er	ppm	453.1	463.5	454.8	457.3	445.0	440.6	455.9	455.4	453.7	464.4	38.9	40.2	36.4	37.4	39.6
Tm	ppm	435.9	441.3	431.5	434.1	420.7	433.2	442.5	435.3	430.4	439.1	36.8	37.5	37.1	36.4	37.0
Yb	ppm	454.6	444.6	449.1	455.7	432.9	453.3	448.4	454.1	441.6	460.4	39.4	39.1	38.5	36.7	39.1
Lu	ppm	435.8	447.4	439.0	439.5	423.4	439.0	450.2	438.9	434.6	440.9	37.6	37.5	36.5	36.0	38.2
Hf	ppm	430.8	441.0	430.0	442.1	430.5	427.4	434.5	439.7	436.6	435.8	40.8	35.6	33.4	32.9	38.9
Ta	ppm	444.9	450.6	448.5	449.4	430.6	446.2	447.8	449.9	442.1	451.6	37.0	36.5	37.2	36.7	37.2
W	ppm	442.6	447.2	442.4	451.8	441.0	443.0	433.1	451.9	445.7	447.3	36.6	38.1	36.5	36.8	38.6
Re	ppm	49.5	49.1	50.0	50.0	50.4	50.9	49.7	50.5	48.6	50.4	6.4	7.6	6.0	7.7	6.9
Pt	ppm	3.0	3.2	2.6	3.8	2.5	4.1	3.1	3.8	3.2	2.9	3.3	2.7	2.6	1.8	2.2
Au	ppm	23.7	23.4	24.3	23.5	23.8	22.8	23.9	23.7	23.6	23.4	4.8	4.4	4.4	4.5	4.8
Tl	ppm	60.5	59.3	57.6	59.8	58.3	61.8	61.2	59.6	58.9	59.4	15.2	14.2	14.6	14.6	15.0
Pb	ppm	422.3	428.4	431.9	427.7	415.7	431.2	423.7	430.6	421.9	427.9	39.8	37.7	37.6	36.8	39.1
Bi	ppm	389.7	376.8	385.9	384.8	381.5	392.1	384.5	387.3	374.9	385.5	35.2	33.3	34.5	33.3	34.2
Th	ppm	453.3	464.0	463.9	446.2	450.2	466.6	455.4	457.7	455.0	458.9	38.6	37.1	36.7	37.0	37.3
U	ppm	457.5	455.8	467.9	469.0	451.9	472.6	459.5	463.1	457.0	458.4	38.7	37.0	37.0	37.3	38.6

Table S5. EMPA data (wt.%) of Sulfides in mafic enclaves from the Beidashan pluton

No.	Analysis point	Mineral	Occurrence	Host mineral
1	KLS16-7-1 FES-3	Pyrrhotite	Inclusion	Clinopyroxene
2	KLS16-7-1 FES-6	Pyrrhotite	Inclusion	Clinopyroxene
3	KLS16-7-1 FES-7	Pyrrhotite	Inclusion	Clinopyroxene
4	KLS16-7-1 FES-5	Pyrrhotite	Inclusion	Clinopyroxene
5	KLS16-7-1 FES-8	Pyrrhotite	Inclusion	Clinopyroxene
6	KLS16-7-10-3-fes	Pyrrhotite	Inclusion	Clinopyroxene
7	KLS16-7-11-2 fes	Pyrrhotite	Inclusion	Clinopyroxene
8	KLS19-2-S1	Pyrrhotite	Inclusion	Clinopyroxene
9	KLS19-2-S2	Pyrrhotite	Inclusion	Clinopyroxene
10	KLS19-1-s2	Pyrrhotite	Inclusion	Clinopyroxene
11	KLS19-1-s3	Pyrrhotite	Inclusion	Clinopyroxene
12	KLS19-1-s4	Pyrrhotite	Inclusion	Clinopyroxene
13	KLS16-9-s0	Pyrrhotite	Inclusion	Clinopyroxene
14	KLS16-9-s1	Pyrrhotite	Inclusion	Clinopyroxene
15	KLS16-9-s3	Pyrrhotite	Inclusion	Clinopyroxene
16	KLS16-9-s8	Pyrrhotite	Inclusion	Clinopyroxene
17	KLS16-9-s10	Pyrrhotite	Inclusion	Clinopyroxene
18	KLS16-9-s11	Pyrrhotite	Inclusion	Clinopyroxene
19	KLS16-9-s12	Pyrrhotite	Inclusion	Clinopyroxene
20	KLS16-9-s13	Pyrrhotite	Inclusion	Clinopyroxene
21	KLS16-9-s14	Pyrrhotite	Inclusion	Clinopyroxene
22	KLS16-2-s1-1	Pyrrhotite	Inclusion	Clinopyroxene
23	KLS16-2-s4-1	Pyrrhotite	Inclusion	Clinopyroxene
24	KLS16-2-s5	Pyrrhotite	Inclusion	Clinopyroxene
25	KLS16-2-s6	Pyrrhotite	Inclusion	Clinopyroxene
26	KLS16-2-s7	Pyrrhotite	Inclusion	Clinopyroxene
27	KLS16-2-s10	Pyrrhotite	Inclusion	Clinopyroxene
28	KLS16-2-s11	Pyrrhotite	Inclusion	Clinopyroxene

No.	Analysis point	Mineral	Occurrence	Host mineral
29	KLS16-2-s12	Pyrrhotite	Inclusion	Clinopyroxene
30	KLS16-2-s14	Pyrrhotite	Inclusion	Clinopyroxene
31	KLS19-1-s1	Pyrite	Intergranular	
32	KLS19-1-s6	Pyrite	Intergranular	
33	KLS16-9-s2	Pyrite	Intergranular	
34	KLS16-9-s5	Pyrite	Intergranular	
35	KLS16-9-s6	Pyrite	Intergranular	
36	KLS19-1-s5	Pyrite	Inclusion	Ilmenite
37	KLS19-1-s8-1	Pyrite	Inclusion	Ilmenite
38	KLS19-1-s8-2	Pyrite	Inclusion	Ilmenite
39	KLS19-1-s7	Pyrite	Inclusion	Ilmenite
40	KLS16-7-1 FES	Pyrite	Inclusion	Ilmenite
41	KLS16-7-1 FES-4	Pyrite	Inclusion	Ilmenite
42	KLS16-2-s1-2	Chalcopyrite	Chalcopyrite exsolution from pyrrhotite	Clinopyroxene
43	KLS16-2-s4-2	Chalcopyrite	Chalcopyrite exsolution from pyrrhotite	Clinopyroxene
44	KLS16-2-s8-2	Chalcopyrite	Intergranular chalcopyrite exsolution from sphalerite	
45	KLS16-2-s13-2	Chalcopyrite	Intergranular mineral cluster	
46	KLS16-2-cpy-1	Chalcopyrite	Intergranular	
47	KLS16-2-cpy-2	Chalcopyrite	Intergranular	
48	KLS16-2-cpy-3	Chalcopyrite	Intergranular	
49	KLS16-2-9.2	Chalcopyrite	Intergranular	
50	KLS16-2-11.1	Chalcopyrite	Intergranular	
51	KLS16-2-12.1	Chalcopyrite	Intergranular	
52	KLS16-2-13.1	Chalcopyrite	Intergranular	
53	KLS16-2-14.2	Chalcopyrite	Intergranular	
54	KLS16-2-sp-3	Arseno-Chalcopyrite	Intergranular	
55	KLS16-2-as-1	Arseno-Chalcopyrite	Intergranular	
56	KLS16-2-as-2	Arsenopyrite	Intergranular	
57	KLS16-2-s8-1	Sphalerite	Intergranular	

No.	Analysis point	Mineral	Occurrence	Host mineral
58	KLS16-2-s13-1	Sphalerite	Intergranular	
59	KLS16-2-sp-1	Sphalerite	Intergranular	
60	KLS16-2-sp-2	Sphalerite	Intergranular	
61	KLS16-2-9.1	Sphalerite	Intergranular	
62	KLS16-2-14.1	Sphalerite	Intergranular	
63	KLS16-2-Pb-1	Galena	Intergranular	
64	KLS16-2-9.3	Galena	Intergranular	
65	KLS16-2-sp-6.1	cobaltite–gersdorffite solid solution	Intergranular	
66	KLS16-2-sp-5.1	cobaltite–gersdorffite solid solution	Intergranular	

Table S5 continued.

No.	Fe	S	Cu	Zn	Pb	As	Co	Ni	Ti	Si	Total
1	61.56	38.06	0.00	0.00	0.00	0.00					99.62
2	61.65	37.98	0.03	0.00	0.03	0.00					99.69
3	59.90	39.40	0.00	0.00	0.01	0.01					99.32
4	62.60	37.01	0.00	0.00	0.03	0.04					99.68
5	61.11	38.36	0.00	0.00	0.02	0.00					99.49
6	60.25	38.89	0.00	0.01	0.00	0.00					99.15
7	62.48	37.01	0.02	0.00	0.00	0.00					99.51
8	60.89	38.63	0.03	0.00	0.02	0.07	0.06	0.06	0.05	0.03	99.84
9	60.77	38.12	0.00	0.01	0.07	0.00	0.06	0.02	0.04	0.16	99.25
10	59.90	38.70	0.07	0.00	0.03	0.01	0.09	0.15	0.03	0.19	99.17
11	59.91	38.96	0.04	0.00	0.05	0.00	0.04	0.01	0.08	0.67	99.76
12	59.80	39.75	0.01	0.00	0.01	0.02	0.07	0.06	0.20	0.07	99.99
13	59.82	39.44	0.00	0.01	0.03	0.09	0.07	0.01	0.01	0.10	99.58
14	60.10	39.31	0.02	0.00	0.07	0.03	0.08	0.02	0.06	0.09	99.78
15	60.64	39.51	0.02	0.00	0.00	0.05	0.11	0.00	0.10	0.05	100.48
16	60.31	39.24	0.00	0.02	0.01	0.10	0.09	0.00	0.02	0.07	99.86
17	60.19	39.65	0.00	0.01	0.06	0.10	0.06	0.00	0.01	0.04	100.12

No.	Fe	S	Cu	Zn	Pb	As	Co	Ni	Ti	Si	Total
18	60.20	39.39	0.02	0.02	0.00	0.01	0.07	0.00	0.00	0.03	99.74
19	60.64	39.19	0.00	0.00	0.01	0.03	0.04	0.02	0.04	0.08	100.05
20	58.84	39.84	0.01	0.01	0.02	0.10	0.08	0.02	0.01	0.40	99.33
21	59.41	39.00	0.29	0.03	0.04	0.01	0.07	0.00	0.03	0.79	99.67
22	60.54	38.24	0.00	0.00	0.02	0.00	0.09	0.01	0.00	0.03	98.93
23	60.38	39.76	0.00	0.02	0.04	0.06	0.10	0.00	0.00	0.00	100.36
24	60.70	38.76	0.00	0.00	0.07	0.09	0.11	0.03	0.02	0.02	99.80
25	60.13	38.99	0.00	0.01	0.04	0.07	0.07	0.00	0.00	0.02	99.33
26	60.13	38.86	0.00	0.01	0.04	0.05	0.06	0.00	0.01	0.03	99.19
27	60.76	38.80	0.00	0.00	0.03	0.06	0.10	0.01	0.00	0.02	99.78
28	59.94	39.50	0.00	0.00	0.04	0.00	0.17	0.03	0.01	0.01	99.70
29	59.97	38.89	0.00	0.00	0.03	0.05	0.11	0.04	0.00	0.04	99.13
30	62.05	37.18	0.01	0.00	0.02	0.03	0.06	0.01	0.03	0.02	99.41
31	45.50	53.35	0.01	0.00	0.00	0.01	0.11	1.06	0.01	0.06	100.11
32	46.10	52.97	0.01	0.01	0.00	0.00	0.07	0.24	0.02	0.12	99.54
33	46.21	53.56	0.01	0.00	0.01	0.10	0.04	0.01	0.19	0.02	100.15
34	46.49	53.39	0.00	0.00	0.02	0.14	0.07	0.00	0.05	0.05	100.21
35	46.12	53.20	0.02	0.00	0.01	0.03	0.07	0.38	0.07	0.04	99.94
36	45.58	53.25	0.00	0.00	0.04	0.05	0.73	0.63	0.85	0.02	101.15
37	45.74	52.57	0.04	0.00	0.05	0.08	0.05	0.12	0.91	0.24	99.80
38	43.84	50.20	6.14	0.03	0.07	0.02	0.05	0.07	0.22	0.00	100.64
39	45.10	52.89	0.34	0.05	0.12	0.07	0.06	0.01	0.21	0.51	99.36
40	46.72	53.13	0.02	0.10	0.00	0.01					99.98
41	46.06	53.95	0.00	0.01	0.02	0.10					100.14
42	45.94	38.71	13.34	0.03	0.02	0.09	0.08	0.01	0.02	0.85	99.09
43	42.01	34.31	17.09	0.26	0.07	0.00	0.06	0.00	0.02	3.25	97.07
44	27.69	34.62	34.63	1.52	0.00	0.00	0.03	0.00	0.08	0.72	99.29
45	26.28	35.28	34.71	4.56	0.00	0.00	0.04	0.00	0.00	0.00	100.87
46	30.06	35.63	33.96	0.03	0.00	0.00	0.22	0.03	0.06	0.01	100.00

No.	Fe	S	Cu	Zn	Pb	As	Co	Ni	Ti	Si	Total
47	30.36	35.51	32.70	0.00	0.00	0.00	0.22	0.05	0.23	0.02	99.09
48	30.04	36.96	33.00	0.02	0.00	0.00	0.18	0.02	0.41	0.00	100.63
49	29.95	35.96	34.03	0.35	0.00	0.00	0.04	0.00	0.00	0.00	100.33
50	29.77	35.85	33.46	0.07	0.02	0.00	0.06	0.00	0.00	0.02	99.25
51	28.24	36.12	32.80	2.20	0.00	0.00	0.05	0.00	0.01	0.02	99.44
52	29.62	35.45	34.45	0.03	0.00	0.00	0.05	0.00	0.01	0.02	99.63
53	29.75	36.09	32.51	1.00	0.00	0.00	0.06	0.00	0.05	0.00	99.46
54	21.77	26.27	17.75	0.00	0.00	26.12	2.71	4.71	0.14	0.57	100.04
55	26.99	35.64	29.72	0.00	0.00	1.25	2.27	4.38	0.23	0.00	100.48
56	45.57	18.47	0.52	0.00	0.12	27.11	4.41	2.40	0.28	0.12	99.00
57	5.28	33.62	4.73	56.24	0.00	0.00	0.05	0.01	0.17	0.04	100.14
58	3.09	33.01	0.71	63.58	0.01	0.05	0.04	0.00	0.03	0.00	100.52
59	6.74	34.90	0.70	56.79	0.01	0.00	0.05	0.00	0.05	0.02	99.26
60	4.20	34.74	0.07	59.89	0.03	0.00	0.05	0.00	0.11	0.01	99.10
61	11.77	33.10	8.64	45.71	0.00	0.00	0.08	0.00	0.05	0.00	99.35
62	4.69	34.42	4.13	57.22	0.00	0.00	0.03	0.00	0.02	0.05	100.56
63	1.98	13.64	2.05	0.68	80.62	0.00	0.01	0.00	0.00	0.00	98.98
64	2.50	13.89	3.21	0.59	79.80	0.00	0.00	0.02	0.00	0.01	100.02
65	15.62	18.57	1.64	0.00	0.06	42.15	5.05	16.70	0.06	0.25	100.10
66	9.04	20.19	0.75	0.00	0.07	44.31	10.49	14.38	0.08	0.03	99.34

Table S6. LA-ICP-MS trace elements concentration (ppm) of sulfides in mafic enclaves

Sample	KLS16-1-1-1	KLS16-1-1-2	KLS16-1-2	KLS16-1-3	KLS16-3-1	KLS16-3-2	KLS16-1-4	KLS16-2-1	KLS16-2-8	KLS16-2-9	KLS16-2-10
Minerals	ccp	ccp	ccp	ccp	ccp	ccp	po	po	po	po	po
Na_2O	45	92	513	70	975	135	2990	642	2389	1137	1573
MgO	1536	13118	7771	2431	51970	24244	136528	33478	112006	54497	64885
Al_2O_3	1827	32526	15260	2171	24226	32503	9081	1791	5779	3618	22090
SiO_2	7683	349842	117270	16410	363099	396243	514045	125436	398604	211071	244892
S	347975	44642	194250	354511	80690	80572	983	318594	84558	217662	208040
K_2O	0	0	755	0	99	6	0	0	110	0	11231
CaO	367	107860	26579	2630	154140	173457	223490	50044	177827	89715	77961
Sc	0	28	6	1	52	4	130	26	138	53	33
TiO_2	60	114432	27719	63	71952	120	2005	489	1700	811	8290
V	6	613	327	11	688	84	242	56	193	96	153
Cr	32	83	648	54	33	20	243	1459	268	484	69
Fe	249923	250433	413027	259923	166273	217507	109938	465689	215951	419326	359747
Co	53	11456	4325	83	4288	2435	71	705	74	329	559
Ni	29	6147	270	29	636	267	3	285	73	147	196
Cu	284985	19518	184196	324412	64200	68842	17	1150	56	704	0
Zn	47123	14250	231	35815	6852	435	179	105	156	188	143
Ga	1.6	4.1	22.1	0.7	8.4	13.6	6.2	1.6	7.8	2.0	7.3
Ge	2.2	14.0	4.2	0.0	8.1	3.3	3.4	3.6	0.0	0.0	0.8
As	84.2	33868.8	6049.7	469.8	9390.7	2369.5	0.4	0.0	10.2	142.8	5.8
Se	508.6	0.0	68.0	37.1	32.3	19.3	28.8	24.0	78.4	5.7	40.3
Nb	0.0	58.2	35.9	0.1	38.4	0.2	0.1	0.1	0.1	0.0	2.4
Ag	244.1	94.4	540.7	390.6	100.3	421.1	0.1	3.9	0.0	3.1	1.2
Cd	152.2	41.5	0.0	147.6	26.8	1.1	0.1	1.7	5.1	0.1	0.3
In	2.8	3.6	0.4	2.4	1.5	0.2	0.3	0.2	0.2	0.3	0.2
Sn	1.1	26.4	17.8	2.6	19.6	2.5	0.0	0.2	0.0	0.8	3.3
Sb	2.9	31.7	15.8	7.4	14.7	12.0	0.0	0.0	0.0	0.0	0.8

Sample	KLS16-1-1-1	KLS16-1-1-2	KLS16-1-2	KLS16-1-3	KLS16-3-1	KLS16-3-2	KLS16-1-4	KLS16-2-1	KLS16-2-8	KLS16-2-9	KLS16-2-10
Te	39.4	22.2	9.0	0.7	0.0	6.8	0.0	10.4	0.0	0.0	1.8
La	0.1	17.9	4.5	0.2	75.4	173.8	12.3	2.9	11.8	5.4	4.1
Hf	0.0	3.9	5.8	0.3	8.6	3.0	2.0	0.2	1.6	0.6	0.8
Ta	0.0	3.3	3.8	0.0	3.6	0.0	0.0	0.0	0.0	0.0	0.1
W	0.1	36.3	9.5	0.2	37.6	0.3	0.1	0.2	0.0	0.1	0.1
Hg	3.7	1.4	0.9	5.6	1.5	1.3	0.6	0.7	0.4	0.0	0.8
Pb	57248.0	728.3	65.1	319.4	59.2	94.6	0.8	1.2	1.5	0.5	66.1
Bi	66.3	3.8	1.0	0.6	0.8	2.6	0.0	0.3	0.3	0.4	0.3
Average EMPA data of host CPX											
SiO_2							517600	517600	517600	517600	517600
Average La-ICP-MS concentration of the host CPX											
Co							39.1	39.1	39.1	39.1	39.1
Ni							17.6	17.6	17.6	17.6	17.6
Cu							0.5	0.5	0.5	0.5	0.5
Zn							104.8	104.8	104.8	104.8	104.8
As							2.1	2.1	2.1	2.1	2.1
Se							1.4	1.4	1.4	1.4	1.4
Ag							0.0	0.0	0.0	0.0	0.0
In							0.1	0.1	0.1	0.1	0.1
Sn							0.0	0.0	0.0	0.0	0.0
Sb							0.1	0.1	0.1	0.1	0.1
Te							0.0	0.0	0.0	0.0	0.0
W							0.0	0.0	0.0	0.0	0.0
Pb							0.3	0.3	0.3	0.3	0.3
Bi							0.0	0.0	0.0	0.0	0.0
Average Cu concentration of iss											
Cu	335120		335122	335123	335124	335125					
Measured SiO_2/CPX SiO_2											

Sample	KLS16-1-1-1	KLS16-1-1-2	KLS16-1-2	KLS16-1-3	KLS16-3-1	KLS16-3-2	KLS16-1-4	KLS16-2-1	KLS16-2-8	KLS16-2-9	KLS16-2-10
							99.31%	24.23%	77.01%	40.78%	47.31%
Measured Cu/iss Cu											
	85.04%		54.96%	96.80%	19.16%	20.54%					
Recaculated trace elements of sulfides											
Co	62.9		7868.7	85.8	22381.0	11852.9		917.8	190.1	529.0	1025.8
Ni	33.7		490.7	29.9	3321.3	1301.1		370.0	257.0	235.9	356.5
Cu	335120.0		335122.0	335123.0	335124.0	335125.0		1517.8	243.0	1189.2	0.0
Zn	47122.5		230.3	35814.2	6851.9	434.7		105.1	326.3	244.6	177.9
As	99.0		11006.8	485.3	49019.5	11534.9		0.0	37.6	239.7	9.2
Pb	67319.2		118.4	329.9	309.2	460.4		1.5	5.6	0.6	125.2
Ag	287.0		983.8	403.5	523.5	2049.9		5.2	0.0	5.3	2.3
Se	598.1		123.7	38.3	168.5	94.1		0.0	336.4	8.7	75.3
Sn	1.3		32.4	2.7	102.4	12.0		0.3	0.0	1.3	6.3
Sb	3.4		28.7	7.6	76.9	58.3		0.0	0.0	0.0	1.4
Te	46.3		16.3	0.7	0.0	33.1		13.7	0.0	0.0	3.4
W	0.1		17.2	0.2	196.2	1.5		0.3	0.0	0.1	0.1
Bi	78.0		1.7	0.6	3.9	12.7		0.3	1.1	0.6	0.5
In	3.3		0.8	2.4	7.9	1.0		0.3	0.7	0.4	0.3

Table S6 continued.

Sample	KLS16-2-11	KLS16-3-3	KLS16-4-1	KLS16-4-2	KLS16-4-3	KLS16-5-1	KLS16-5-2	KLS16-6-1	KLS16-6-2	KLS16-6-3
Minerals	po	po	po	po	po	po	po	po	po	po
Na_2O	1678	1063	893	1992	799	3220	1567	1678	3328	218
MgO	85303	52516	52582	58390	63194	61766	68267	85821	54619	4618
Al_2O_3	6766	4833	3537	4022	3060	11662	6134	5415	17568	2162
SiO_2	339483	226808	214097	246179	324360	318863	279050	339201	189351	47444
S	128613	218453	211420	202034	157878	168763	178577	128390	240764	449796
K_2O	128	135	19	18	0	3701	485	0	1340	94

Sample	KLS16-2-11	KLS16-3-3	KLS16-4-1	KLS16-4-2	KLS16-4-3	KLS16-5-1	KLS16-5-2	KLS16-6-1	KLS16-6-2	KLS16-6-3
CaO	143369	96543	84457	106525	120314	101880	113681	144506	70097	13379
Sc	92	38	47	79	62	58	62	125	35	3
TiO_2	1694	1086	883	1197	1288	1341	1123	1391	4958	363
V	168	88	85	140	294	131	114	160	148	15
Cr	172	563	333	289	62	578	198	453	1076	249
Fe	291417	392795	428947	376137	325853	324056	348381	292060	413438	470029
Co	319	462	894	202	545	296	408	295	682	4512
Ni	148	71	361	179	253	179	189	65	240	3518
Cu	416	2216	1302	1747	1703	3174	1584	237	2156	2534
Zn	168	254	103	170	233	149	153	176	165	36
Ga	2.2	5.0	2.2	5.0	3.8	3.9	3.3	3.6	5.4	0.1
Ge	1.0	2.8	0.8	4.8	1.9	1.1	1.2	3.8	2.5	0.9
As	10.7	3.1	0.0	618.1	58.7	141.7	2.7	0.0	2.0	882.8
Se	30.5	0.0	20.2	29.8	10.7	5.3	0.6	0.0	0.0	14.2
Nb	0.1	0.0	0.0	20.9	0.0	0.5	0.0	0.0	1.8	0.1
Ag	2.8	2.3	4.2	3.6	1.2	3.5	6.3	0.9	3.4	12.0
Cd	0.0	3.4	1.0	0.0	1.0	2.0	0.0	0.3	5.9	0.3
In	0.2	0.4	0.2	0.2	0.3	0.5	0.2	0.3	0.2	0.1
Sn	0.2	1.2	0.4	0.9	1.3	1.8	0.6	0.9	1.2	0.8
Sb	0.0	0.1	0.0	0.1	0.1	0.2	0.0	0.1	0.0	7.0
Te	1.6	3.0	2.0	3.3	3.4	0.0	0.0	0.0	0.0	2.7
La	12.3	12.6	5.7	10.4	16.5	14.4	9.2	11.1	8.2	24.4
Hf	2.4	1.2	1.0	1.6	1.9	1.2	1.3	2.4	1.2	0.0
Ta	0.0	0.1	0.0	0.0	0.0	0.1	0.0	0.0	0.1	0.0
W	0.4	0.0	0.0	0.3	0.1	0.2	0.0	0.1	0.3	0.3
Hg	0.0	11.6	0.6	0.1	0.3	0.1	0.7	1.3	0.0	0.4
Pb	0.3	2030.3	0.7	0.5	0.7	4.6	1.2	0.8	0.8	80.5
Bi	0.0	0.3	0.3	0.1	0.2	1.0	0.4	0.1	0.1	6.0

Average EMPA concentration of host CPX

Sample	KLS16-2-11	KLS16-3-3	KLS16-4-1	KLS16-4-2	KLS16-4-3	KLS16-5-1	KLS16-5-2	KLS16-6-1	KLS16-6-2	KLS16-6-3
SiO_2	517600	517600	517600	517600	517600	517600	517600	517600	517600	517600
Average La-ICP-MS concentration of the host CPX										
Co	39.1	39.1	39.1	39.1	39.1	39.1	39.1	39.1	39.1	39.1
Ni	17.6	17.6	17.6	17.6	17.6	17.6	17.6	17.6	17.6	17.6
Cu	0.5	0.5	0.5	0.5	0.5	0.5	0.5	0.5	0.5	0.5
Zn	104.8	104.8	104.8	104.8	104.8	104.8	104.8	104.8	104.8	104.8
As	2.1	2.1	2.1	2.1	2.1	2.1	2.1	2.1	2.1	2.1
Se	1.4	1.4	1.4	1.4	1.4	1.4	1.4	1.4	1.4	1.4
Ag	0.0	0.0	0.0	0.0	0.0	0.0	0.0	0.0	0.0	0.0
In	0.1	0.1	0.1	0.1	0.1	0.1	0.1	0.1	0.1	0.1
Sn	0.0	0.0	0.0	0.0	0.0	0.0	0.0	0.0	0.0	0.0
Sb	0.1	0.1	0.1	0.1	0.1	0.1	0.1	0.1	0.1	0.1
Te	0.0	0.0	0.0	0.0	0.0	0.0	0.0	0.0	0.0	0.0
W	0.0	0.0	0.0	0.0	0.0	0.0	0.0	0.0	0.0	0.0
Pb	0.3	0.3	0.3	0.3	0.3	0.3	0.3	0.3	0.3	0.3
Bi	0.0	0.0	0.0	0.0	0.0	0.0	0.0	0.0	0.0	0.0
Average EMPA data of iss										
Cu										
Measured SiO_2/CPX SiO_2										
	65.59%	43.82%	41.36%	47.56%	62.67%	61.60%	53.91%	65.53%	36.58%	9.17%
Measured Cu/iss Cu										
Recaculated trace elements of sulfides										
Co	852.9	792.0	1496.4	350.6	1394.6	706.9	839.4	781.9	1053.6	4963.5
Ni	397.4	112.1	603.3	326.0	646.9	439.2	390.0	156.1	368.0	3870.8
Cu	1208.1	3943.9	2220.6	3331.1	4561.0	8264.9	3437.2	688.0	3399.0	2789.7
Zn	289.1	369.9	101.8	229.8	448.5	218.7	208.9	311.5	200.3	28.6
As	27.1	0.0	0.0	1.0	153.9	365.8	3.4	0.0	1.9	971.7
Pb	0.1	3613.6	0.9	0.7	1.4	11.5	2.3	1.7	1.1	88.6

Sample	KLS16-2-11	KLS16-3-3	KLS16-4-1	KLS16-4-2	KLS16-4-3	KLS16-5-1	KLS16-5-2	KLS16-6-1	KLS16-6-2	KLS16-6-3
Ag	8.3	4.2	7.2	7.0	3.3	9.2	13.7	2.7	0.0	13.2
Se	86.0	0.0	0.0	1.0	26.3	0.0	0.0	0.0	0.0	0.0
Sn	0.6	2.2	0.6	1.7	3.4	4.7	1.3	2.5	1.9	0.9
Sb	0.0	0.0	0.0	1.0	0.2	0.5	0.0	0.2	0.0	7.7
Te	4.6	5.3	3.4	6.2	9.2	0.0	0.0	0.0	0.0	3.0
W	1.2	0.0	0.0	0.5	0.2	0.5	0.0	0.3	0.4	0.3
Bi	0.0	0.5	0.4	0.1	0.5	2.5	0.8	0.3	0.1	6.6
In	0.4	0.6	0.3	0.3	0.7	1.2	0.3	0.7	0.3	0.1

Other Supplementary Materials for this manuscript include the following:

Supplementary Data S1 to S2 (separate file)

Data S1. LA-ICP-MS trace elements concentration (ppm) of sulfide standards, and errors.

Data S2. LA-ICP-MS trace elements detection limit of sulfide inclusions in mafic enclaves
